# Comparisons of plasma aldosterone and renin data between an automated chemiluminescent immunoanalyzer and conventional radioimmunoassays in the screening and diagnosis of primary aldosteronism

**DOI:** 10.1371/journal.pone.0253807

**Published:** 2021-07-09

**Authors:** Naohisa Tamura, Erika Watanabe, Rumi Shirakawa, Eiji Nakatani, Kanako Yamada, Hiroshi Hatakeyama, Mizuki Torii-Hanakita, Chika Kyo, Rieko Kosugi, Tatsuo Ogawa, Masato Kotani, Takeshi Usui, Tatsuhide Inoue

**Affiliations:** 1 Research Support Center, Shizuoka General Hospital, Shizuoka, Shizuoka, Japan; 2 Center for Diabetes, Endocrinology and Metabolism, Shizuoka General Hospital, Shizuoka, Shizuoka, Japan; 3 Department of Clinical Laboratory Medicine, Shizuoka General Hospital, Shizuoka, Shizuoka, Japan; 4 Department of Medical Genetics, Shizuoka General Hospital, Shizuoka, Shizuoka, Japan; Max Delbruck Centrum fur Molekulare Medizin Berlin Buch, GERMANY

## Abstract

Determining values of plasma renin activity (PRA) or plasma active renin concentration (ARC), plasma aldosterone concentration (PAC), and aldosterone-to-renin ratio (ARR) is essential to diagnose primary aldosteronism (PA), but it takes several days with conventional radioimmunoassays (RIAs). Chemiluminescent enzyme immunoassays for PAC and ARC using the Accuraseed^®^ immunoanalyzer facilitated the determination, but relations between Accuraseed^®^ immunoanalyzer-based and RIA-based values in samples of PA confirmatory tests and adrenal venous sampling remained to be elucidated. We addressed this issue in the present study. This is a prospective, cross-sectional study. ARC and PAC values were measured by the Accuraseed^®^ immunoanalyzer in samples, in which PRA and PAC values had been measured by the PRA-FR^®^ RIA and SPAC^®^-S Aldosterone kits, respectively. The relations between Accuraseed^®^ immunoanalyzer-based and RIA-based values were investigated with regression analyses. The optimal cutoff of Accuraseed^®^ immunoanalyzer-based ARR for PA screening was determined by the receiver operating characteristic analysis. After log-log transformations, linear relations with high coefficients of determination were observed between Accuraseed^®^ immunoanalyzer-based and RIA-based data of renin and aldosterone. Following the PA guidelines of Japan Endocrine Society, Accuraseed^®^ immunoanalyzer-based cutoffs were calculated from the regression equations: the basal PAC for PA screening >12 ng/dL, PAC for the saline infusion test >8.2 ng/dL, ARC for the furosemide-upright test <15 pg/mL, and ARR for the captopril challenge test >3.09 ng/dL per pg/mL. The optimal cutoff of Accuraseed^®^ immunoanalyzer-based ARR for PA screening was >2.43 ng/dL over pg/mL not to overlook bilateral PA patients. The present study provided conversion formulas between Accuraseed^®^ immunoanalyzer-based and RIA-based values of renin, aldosterone, and ARR, not only in basal samples but also in samples of PA confirmatory tests and adrenal venous sampling. Although validation studies are awaited, the present study will become priming water of harmonization of renin and aldosterone immunoassays.

## Introduction

Primary aldosteronism (PA) is a common form of secondary hypertension with increased cardiovascular morbidity [1–7]. The simultaneous measurements of plasma aldosterone concentration (PAC), and plasma renin activity (PRA) or plasma active renin concentration (ARC) values, and the calculation of aldosterone-to-renin ratio (ARR) values are the recommended screening procedure for PA [8–10]. In most institutions, these values are measured by radioimmunoassay (RIA) kits, and the PA guidelines 2009 of Japan Endocrine Society (JES) adopted the SPAC^®^-S Aldosterone kit (Fujirebio Inc., Tokyo, Japan) as the standard kit to measure PAC values [9]. However, the fact that it takes several days to obtain the data by the RIA kits makes completing the PA screening at the first visit impossible and causes delay in PA diagnosis. Ideally, PAC and PRA values should be measured after the discontinuation of medications affecting these values; the duration of discontinuation is recommended >4 weeks for mineralocorticoid receptor antagonist and diuretics, and >2 weeks for angiotensin converting enzyme inhibitors, angiotensin II receptor antagonist, and β-blockers [11]. If PAC and PRA values could be obtained at the first visit and the screening of PA could be completed before the beginning of anti-hypertension medications, we would not be bothered with the influences of medications [11]. This is important because medications recognized as less affecting PAC and PRA values actually, significantly affect these values [12]. The rapid PAC and PRA measurements would enable us to distinguish secondary aldosteronism from PA at the first visit; patients with secondary aldosteronism share the resistant hypertension and hypokalemia with PA patients as the clinical presentation, but require different approaches for screening, diagnosis, and treatment [10]. Switching anti-hypertensive medications may result in the deterioration of patient conditions and intensified medical follow up is required while medications are switched [11]; the rapid PAC and PRA measurements shorten the duration of this dangerous period. Recently, chemiluminescent enzyme immunoassay (CLEIA) kits for PAC and ARC (the Accuraseed^®^ Aldosterone and Accuraseed^®^ Renin kits, FUJIFILM Wako Pure Chemical Corporation, Osaka, Japan) were developed. With the Accuraseed^®^ automated chemiluminescent enzyme immunoanalyzer (FUJIFILM Wako Pure Chemical Corporation, Osaka, Japan), these CLEIA kits enabled us to obtain PAC and ARC data within 1 h after blood collection, facilitating the PA screening.

It was reported that the cross-reactivities of PAC immunoassays on 18-oxocortisol and 18-hydroxycortisol might influence PAC data in adrenal vein (AdV) samples with high PAC levels [13]. PAC immunoassays could cross-react also with 18-hydroxycorticosterone, and serum 18-hydroxycorticosterone to aldosterone ratios were reported to increase beyond 70 in samples at the end of saline infusion test (SIT) from low-renin essential hypertension (EHT) patients [14]. It was also reported that PAC values measured by immunoassays were different from those measured by the liquid chromatography-tandem mass spectrometry [13, 15, 16], and the serum certified reference material of aldosterone (NMIJ CRM 6402) was established to standardize PAC measurements in Japan [13]. JES issued the second operation guideline for the standardization of aldosterone measurements in daily clinical practice on February 18, 2020 [17] and recommended us to determine the regression equation between PAC values measured by the SPAC^®^-S Aldosterone kit and those measured by another kit after calibration with NMIJ CRM 6402 and to convert another-kit-based PAC values to SPAC^®^-S Aldosterone kit-based PAC values by the determined regression equation, in order to maintain the current clinical judgment values. Therefore, clinical judgment values may differ from those described in the current Japanese PA guidelines, if PAC values measured by a kit other than the SPAC^®^-S Aldosterone kit are used without conversion to SPAC^®^-S Aldosterone kit-based values. Actually, when the JES’s PA guidelines 2009 were developed, there was a problem that PAC cutoffs for SIT differed between the SPAC^®^-S Aldosterone kit and Aldosterone RIA kit II^®^ (Dynabot, Tokyo, Japan). In order to settle this problem, the formula to convert Aldosterone RIA kit II^®^-based PAC values to SPAC^®^-S Aldosterone kit-based values was included in the JES’s PA guidelines [9]. Problems like this are common in hormone immunoassays, and the harmonization of measurements with different immunoassays have been done for many hormones [18–21].

It was reported that PAC values measured by the Accuraseed^®^ Aldosterone kit (hereafter abbreviated as CLEIA-PAC) showed a high-degree positive correlation with those measured by the SPAC^®^-S Aldosterone kit (hereafter abbreviated as RIA-PAC) [15]. ARC values measured by the Accuraseed^®^ Renin kit (hereafter abbreviated as CLEIA-ARC) reportedly showed high-degree positive correlations not only with ARC values measured by an immunoradiometric assay kit (the Renin IRMA-FR^®^ kit, Fujirebio Inc., Tokyo, Japan) but also with PRA values measured by an RIA kit (the Renin RIABEAD^®^ kit) [15]. The Accuraseed^®^ Aldosterone and Accuraseed^®^ Renin kits have obtained pharmaceutical approval and are already used in clinical practice in Japan. However, the relations between Accuraseed^®^ immunoanalyzer-based and RIA-based values of renin and aldosterone in the samples of PA confirmatory tests and the adrenal venous sampling (AVS) have not been clarified. Moreover, the linear regression equation between CLEIA-PAC (*y* [ng/dL]) and RIA-PAC (*x* [ng/dL]) values was reported to be *y* = 1.042*x* + 1.478 [15]. The PAC cutoff values for the PA screening, captopril challenge test (CCT), and SIT are 5~12 ng/dL [8–10]. If the same, reported relation would be observed in the samples of CCT and SIT, the *y*-intercept would have a significant impact on the cutoffs. Therefore, we investigated the relations between Accuraseed^®^ immunoanalyzer-based and conventional RIA-based values of renin and aldosterone not only in basal conditions but also in loading tests and AVS in the present study and obtained the regression equations for the samples of basal conditions, loading tests, and AVS.

## Materials and methods

### Study design and participants

This is a prospective, cross-sectional study. Patients, who admitted or attended to the Center for Diabetes, Endocrinology and Metabolism, Shizuoka General Hospital from April 1, 2018 to March 31, 2019 and received blood sampling for the measurements of PAC or PRA values by conventional RIA kits (the SPAC^®^-S Aldosterone and PRA-FR^®^ RIA kits, Fujirebio Inc., Tokyo, Japan) [22, 23], were consecutively invited to the present study. Inclusion criteria were as follows: 1) patients for whom PAC or PRA values were measured by the conventional RIA kits described above, 2) patients 20-yr-old or older, 3) patients from whom informed consent for the study was obtained in documents upon information disclosure of the study. The exclusion criterion was as follows: 1) patients who withdrew the informed consent for the study. Patients who matched all inclusion criteria and did not match the exclusion criterion were included in the study. The study size of present study was set to be 300 as the number of samples that was expected to be obtained during the study period described above. The protocol of this study was approved by the Clinical Research Ethics Committee of Shizuoka General Hospital (SGHIRB#2017075) and performed following the Code of Ethics of the World Medical Association (1964 Declaration of Helsinki and its later amendments).

### Blood sampling

In the basal condition, blood samples were withdrawn from the antecubital veins of participants into collection tubes with disodium ethylenediaminetetraacetate dihydrate after 30-min bed rest in the supine position. For some patients, blood samples from the peripheral veins were obtained in ambulatory conditions. CCT, SIT, the furosemide-upright test (FUP), and AVS were performed following the JES’s PA guidelines 2009 [9]. In CCT, SIT, and FUP, at first, blood samples before loading were obtained from the peripheral veins of patients after 30-min bedrest in the supine position. In CCT, patients were kept in the supine position during the test, and blood samples were collected from the peripheral veins 60 and 90 min after the per os administration of 50-mg captopril. In SIT, blood samples were collected from the peripheral veins after the intravenous (iv) administration of 2-L saline over 4 h in the supine position. In FUP, after the iv administration of 40-mg furosemide, patients were kept standing or walking for 2 h, and then blood samples were collected from the peripheral veins in the sitting position. For AVS, a catheter was advanced through a sheath, which was inserted into the right femoral vein, to the inferior vena cava (IVC) inferior to the confluence of IVC and AdVs, and left and right AdVs sequentially, and blood samples were collected through the catheter. Fifteen through 45 min after the iv administration of 0.25-mg tetracosactide, the synthetic peptide identical to the 24-amino acid segment at the N-terminal of adrenocorticotropic hormone (ACTH), the blood sampling from IVC and bilateral AdVs were repeated. The rapid ACTH test was carried out, as reported previously [24]. Blood samples before loading were obtained from the peripheral veins of patients after 30-min bedrest in the supine position. Patients were kept in the supine position during the test and blood samples were collected from the peripheral veins 30 and 60 min after the iv administration of 0.25-mg tetracosactide. Blood samples collected in the basal and ambulatory conditions, before loading in CCT, SIT, FUP, and the rapid ACTH test, 60 min after the loading of CCT (CCT60), at the end of SIT and FUP (post-SIT and post-FUP, respectively), and 30 and 60 min after the loading of rapid ACTH test (post-ACTH), and those collected from IVC and bilateral AdVs before and after the ACTH loading in AVS were used in the present study. Only samples collected after the informed consent for the study had been obtained were used in the analyses.

### Assays

The SPAC^®^-S Aldosterone and PRA-FR^®^ RIA kits (Fujirebio Inc., Tokyo, Japan) [22, 23] were selected as the reference methods to measure PAC and PRA values, because the former had been adopted as the standard method to measure PAC values in JES’s PA guidelines and the latter was commonly used in daily clinical practice in Japan. Upon blood collection, plasma was isolated and divided into two parts; one part was sent to a laboratory of SRL Inc., Tokyo, Japan to measure RIA-PAC and PRA values with the reference methods (The SPAC^®^-S Aldosterone and PRA-FR^®^ RIA kits) following manufacturer’s instructions [22, 23] as daily clinical practice. For PAC measurements, aldosterone standards or plasma samples were put into tubes coated with anti-aldosterone antibody with fixed-dose ^125^I-labelled aldosterone. After incubation with shaking at 200 rpm at 15~30 °C for 3 h, the supernatant was removed, and the radioactivity remained in the tubes was detected. PAC values of samples were determined by the comparisons of radioactivity between tubes that had contained the samples and standards. The intra-assay coefficient of variation (*CV*) of this PAC assay kit claimed by the manufacturer is <20%. As reference data, intra-assay *CV* values for samples with 10.3, 33.6, and 73.2-ng/dL PAC values are claimed to be 8.3%, 3.9%, and 1.8%, respectively [22]. For PRA measurements, plasma samples were incubated with the angiotensin I generation buffer at 37 °C for 90 min. The samples after the angiotensin I generation from angiotensinogen that had been contained in the samples or angiotensin I standards were incubated with fixed-dose ^125^I-labelled angiotensin I and the anti-angiotensin I antibody at 2~8 °C for 16~24 h. The immunocomplex was precipitated by incubation with the second antibody and polyethylene glycol at 2~8 °C for 1 h and centrifugation at 2,000x g for 20 min at 2~8 °C, and the radioactivity of pellets was detected. The concentrations of generated angiotensin I of samples were determined by the comparison of radioactivity between pellets derived from the samples and standards, and PRA values were calculated from the generated angiotensin I concentrations. The intra-assay *CV* of this PRA assay kit claimed by the manufacturer is <10% [23]. The cross-reactivities of the SPAC^®^-S Aldosterone and PRA-FR^®^ RIA kits are shown in S1 Table.

The other part of plasma was stored frozen at -80 °C. When the data of RIA-PAC and PRA were fixed, frozen samples were thawed and their CLEIA-PAC and CLEIA-ARC values were measured by the Accuraseed^®^ Aldosterone and Accuraseed^®^ Renin kits [15] as the test methods, respectively, following manufacturer’s instructions. We measured CLEIA-PAC values in samples, in which RIA-PAC values had been measured, and CLEIA-ARC values in samples, in which PRA values had been measured (Table 1). Where RIA-PAC or CLEIA-PAC values were greater than the upper limit of quantitation (upper LoQ, 160 ng/dL), appropriately diluted samples were used in assays and data were calculated by multiplying measured values by dilution factors. Samples with RIA-PAC values less than the lower LoQ (2.5 ng/dL) were excluded from PAC analyses. There were no samples with PRA or CLEIA-ARC values greater than the upper LoQ (40 ng/mL as angiotensin I or 500 pg/mL, respectively). Samples with PRA values lower than the lower LoQ (0.2 ng/mL/h) were excluded from renin analyses. As described in the Results section, samples with the values of CLEIA-PAC in the first assays (CLEIA-PAC-1st) being <20 ng/dL were subjected to re-assays to obtain more accurate, final values (CLEIA-PAC-final). RIA-based ARR (RIA-ARR) was calculated by dividing RIA-PAC by PRA, and Accuraseed^®^ immunoanalyzer-based ARR (CLEIA-ARR) was calculated by dividing CLEIA-PAC-final by CLEIA-ARC. Samples, for which PRA, CLEIA-ARC, RIA-PAC, or CLEIA-PAC-final values were out of LoQs, were excluded from ARR analyses. Even where two or more samples were obtained from each of some participants, data from all samples were used as separate data unless otherwise stated, since the primary purpose of the present study was to evaluate the relations of measured values between the CLEIA and conventional RIA kits.

**Table 1:** Numbers and classifications of samples.

diagnoses	non-PA		PA		group of sampling conditions	
kinds of measured values	PRA, CLEIA-ARC	RIA-PAC, CLEIA-PAC	PRA, CLEIA-ARC	RIA-PAC, CLEIA-PAC	PRA, CLEIA-ARC	RIA-PAC, CLEIA-PAC
ambulatory	3 (3) [0]	3 (3) [0]	0 (0) [0]	0 (0) [0]	Basal	Basal
basal	26 (26) [1]	22 (22) [5]	9 (9) [0]	9 (9) [0]		
before loading	12 (11) [1]	9 (8) [4]*	22 (21) [3]	25 (24) [0]		
IVC samples before the ACTH loading in AVS	N.A.	N.A.	13 (13) [2]	16^†^ (15) [0]		
CCT60	8 (7) [0]	5 (5) [3]	23 (23) [2]	22 (22) [3]	RStim	ASup
post-SIT	1 (1) [0]	0 (0) [1]*	8 (8) [4]	12 (12) [0]	Post-SIT	
post-FUP	3 (3) [0]	3 (3) [0]	12 (12) [0]	12 (12) [0]	RStim	AStim
post-ACTH	N.A.	9 (5) [0]	N.A.	0 (0) [0]	N.A.	
IVC samples after the ACTH loading in AVS	N.A.	N.A.	N.A.	16 (15) [0]	N.A.	
adrenal vein (AdV) samples before and after the ACTH loading in AVS	N.A.	N.A.	N.A.	58 (15) [0]	N.A.	AdV
Basal sum	41 (37) [2]	34 (32) [9]	44 (37) [5]	50^†^ (40) [0]		
RStim sum	11 (7) [0]	N.A.	35 (23) [2]	N.A.		
Post-SIT sum	1 (1) [0]	N.A.	8 (8) [4]	N.A.		
ASup sum	N.A.	5 (5) [4]	N.A.	34 (24) [3]		
AStim sum	N.A.	12 (8) [0]	N.A.	28 (24) [0]		
AdV sum	N.A.	N.A.	N.A.	58 (15) [0]		
Total	53 (37) [2]	51 (33) [13]	87 (37) [11]	170 (40) [3]		

The numbers of samples that were used in statistical analyses on renin (PRA and CLEIA-ARC) and aldosterone (RIA-PAC and the final values of CLEIA-PAC) are shown divided by sampling conditions and diagnoses. The numbers of patients, from whom samples used in the above analyses were obtained, are shown in parentheses. The numbers of samples that were not used in the above analyses are shown in brackets; PRA or RIA-PAC values, or the final values of CLEIA-PAC of these samples were less than the lower limit of quantitation (LoQ) of each assay. Patient numbers in sum and total rows are smaller than sums of the rows looked up, where samples of different sampling conditions were obtained from some patients. How sampling conditions were grouped for statistical analyses is also shown at the right end. AVS: adrenal venous sampling. CCT60: 60 min after the loading of captopril challenge test. Post-SIT: at the end of saline infusion test. Post-FUP: at the end of furosemide-upright test. Post-ACTH: 30 and 60 min after the loading of rapid adrenocorticotropic hormone test. RStim: renin stimulation test. ASup: aldosterone suppression test. AStim: aldosterone stimulation test. N.A.: not applicable.

*For the analyses of CLEIA-PAC-1st values, samples shown in brackets were also used except for one sample each with RIA-PAC being less than the lower LoQ.

^†^Two IVC samples before the ACTH loading in AVS were obtained from a PA patient and they were used only for PAC analyses.

In order to reconfirm the precision of Accuraseed^®^ Aldosterone kit, samples with known aldosterone concentrations (6, 9, 12, 15, 40, and 80 ng/dL) were prepared by diluting aldosterone (A9477, CAS Registry Number 52-39-1, Sigma-Aldrich Co. LLC., MO, USA) with the charcoal stripped serum prepared from human plasma (Human serum, double charcoal stripped, delipidized, SP1070, Golden West Biologicals, Inc., CA, USA) were measured in quintuplicate with the kit and the intra-assay precision was determined. We also measured PAC values of ten patient samples, which had shown CLEIA-PAC-1st values ≥20 ng/dL, in triplicate and evaluated the *CV* value for each sample.

### Groups of sampling conditions

Sampling conditions were grouped for statistical analyses because sample numbers were too small in some conditions (Table 1). The Basal group consisted of ambulatory, basal, and before loading samples, and IVC samples before the ACTH loading in AVS for renin and PAC analyses. For renin analyses, the renin stimulation test (RStim) group consisted of CCT60 and post-FUP samples, and the Post-SIT group consisted of post-SIT samples. For PAC analyses, the aldosterone suppression test (ASup) group consisted of CCT60 and post-SIT samples; the aldosterone stimulation test (AStim) group consisted of post-FUP and post-ACTH peripheral samples, and IVC samples after the ACTH loading in AVS; the AdV group consisted of AdV samples before and after the ACTH loading in AVS. Clinical data including diagnoses, medical histories, and other laboratory data were collected from participants’ medical records. PA was screened for and diagnosed based on PRA, RIA-PAC, and RIA-ARR values, following the JES’s PA guidelines and the consensus statement on clinical practice on PA of JES and the Japan Association of Endocrine Surgeons [9, 25].

### Statistical analyses

The normality of variables was tested by the D’Agostino-Pearson test. For variables that are normally distributed, the significance of differences between the means of two groups was assessed by the unpaired *t*-test, and the Pearson’s correlation and linear regression analyses were performed. For variables that are not normally distributed, the significance of differences between the medians of two groups was assessed by the Mann-Whitney U test and Spearman’s rank correlation test was performed for correlation analyses. If both untransformed and log-transformed values did not pass the normality test, the likelihoods of normal and log-normal distributions were compared as described by Burnham and Anderson [26]. Variables, for which the likelihood of log-normal distribution is higher than that of normal distribution, were analyzed as they had been log-normally distributed. For variables, which are log-normally distributed, log-transformed values were used for the regression analysis. The agreement between CLEIA-PAC and RIA-PAC values was analyzed with the Bland-Altman analysis. The lower LoQ of CLEIA-PAC was determined by the non-linear regression equation between *CV* values of CLEIA-PAC and CLEIA-PAC-1st values as *CV* was ≤20%. The optimal cutoff of CLEIA-ARR for the screening of PA was determined by the receiver operating characteristic (ROC) analysis as the Youden indices were maximized [27]. Fisher’s exact test was performed for assessing the independence between two binary variables. The significance level was set as *p* <0.05. Statistical analyses were performed with Prism Ver. 9.0.0 (Graphpad Software Inc., San Diego, CA).

## Results

### Participants and samples

**Fig 1:**
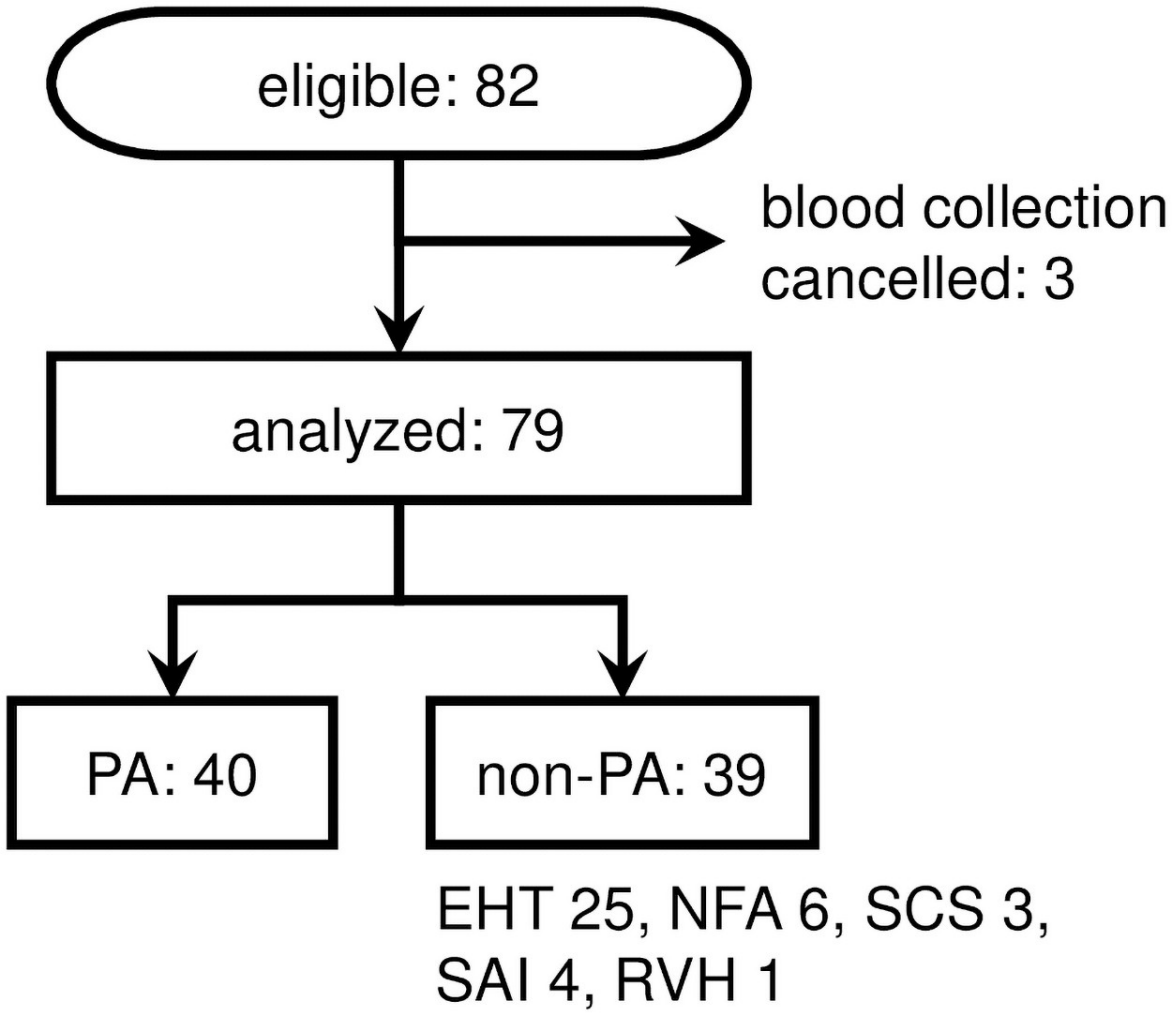
Study flow chart. PA: primary aldosteronism. EHT: essential hypertsnsion. NFA: non-functioning adrenal tumor without EHT. SCS: subclinical Cushing’s syndrome. SAI: secondary adrenal insufficiency. RVH: renovascular hypertension.

Informed consent for participation in this study was obtained from 82 eligible patients, and plasma samples from 79 patients, excluding three patients, for whom the blood collection was canceled, were used. Participants consisted of 40 PA and 39 non-PA patients (Fig 1). Non-PA patients consisted of 25 EHT patients, six non-functioning adrenal tumor patients without EHT, three subclinical Cushing’s syndrome (SCS) patients, four secondary adrenal insufficiency (SAI) patients, and one renovascular hypertension patient (Fig 1).

The clinical characteristics of participants are shown in Table 2. The sex proportion was not different between the two groups (*p* = 0.264). In the PA group, age and basal PRA values were lower, and K supplementation doses and basal RIA-PAC and RIA-ARR values were higher than those in the non-PA group (Table 2). There were no differences in other parameters between the two groups (Table 2).

**Table 2:** Clinical characteristics of participants.

diagnoses	non-PA	PA	*p* values	*DF*	Mann-Whitney *U*
participant numbers	39	40	N.A.	N.A.	N.A.
female/male	17/22	23/17	0.264	1	N.A.
age (yr)	57.8 (13.8)	51.4 (11.6)	0.027*	77	N.A.
height (cm)	161.3 (9.1)	162.2 (8.9)	0.654	77	N.A.
weight (kg)	63.6 [56.0, 69.9]	64.6 [58.5, 82.4]	0.212	N.A.	652
BMI (kg/m^2^)	24.4 [21.8, 27.6]	25.6 [22.9, 29.0]	0.131	N.A.	625.5
SBP (mmHg)	140.4 (26.1)	150.0 (16.2)	0.053	77	N.A.
DBP (mmHg)	85.0 (23.4)	93.2 (14.2)	0.063	77	N.A.
number of anti-HTN drugs^†^	1.0 [0.0, 3.0]	1.0 [1.0, 2.0]	0.402	N.A.	696.5
eGFR (mL/min/1.73 m^2^)	76.0 [63.0, 91.0]	78.7 [72.4, 92.3]	0.378	N.A.	689.5
plasma K^+^ (mEq/L)	4.0 (0.4)	3.8 (0.5)	0.072	77	N.A.
K supplementation (mEq/day)	0.0 [0.0, 0.0]	0.0 [0.0, 3.0]	0.021*	N.A.	626.5
basal PRA (ng/mL/h) ^‡^^,^ ^§^	1.20 [0.55, 2.20]	0.40 [0.30, 0.50]	<0.0001*	N.A.	197
basal RIA-PAC (ng/dL) ^‡^	9.55 [6.95, 13.28]	13.90 [9.49, 22.50]	0.0005*	N.A.	430
basal RIA-ARR (ng/dL over ng/mL/h) ^‡^^,^ ^§^	8.08 [4.49, 15.93]	34.33 [23.33, 51.27]	<0.0001*	N.A.	81

Variables that are normally distributed are shown as “mean (standard deviation)” with the values of degree of freedom (*DF*) and *p* values for the t test, and those that are not normally distributed are shown as “median [25th, 75th percentiles]” with *U* and *p* values for the Mann-Whitney U test. PA: primary aldosteronism. N.A.: not applicable. BMI: body mass index. SBP: systolic blood pressure. DBP: diastolic blood pressure. eGFR: estimated glomerular filtration rate. K: potassium.

*p <0.05.

^†^Sums of anti-hypertension (HTN) drug doses divided by usual doses.

^‡^The results of analyses on the samples of Basal group are shown. The rank orders of sampling conditions are set as (1) basal, (2) ambulatory, (3) before loading, and (4) IVC samples before the ACTH loading in AVS. The result of sample of the highest rank order is used if multiple samples were obtained from the same patient. If two samples of the same rank existed for the same patient, the sample obtained on earlier date was used.

^§^Data of 37 non-PA and 37 PA patients, whose basal PRA values were not less than the lower limit of quantitation, were analyzed.

The numbers of samples and cases, from whom the samples were obtained, in subsets divided by diagnoses, sampling conditions, and the kinds of parameters analyzed are shown in Table 1. PRA values were measured in 153 samples, but 13 samples with PRA values being less than the lower LoQ were excluded from renin analyses: two samples from two non-PA patients (EHT and SAI), and eleven samples from three PA patients. The PRA and CLEIA-ARC values of the other 140 samples from 37 non-PA and 37 PA patients were analyzed (Table 1). On the other hand, RIA-PAC values were measured in 237 samples, but two samples with RIA-PAC values being less than the lower LoQ were excluded: one before loading sample from a SAI patient and one post-SIT sample from a SCS patient (Table 1). The remaining 235 samples from 39 non-PA and 40 PA patients were used to analyze the relation between RIA-PAC and CLEIA-PAC-1st values. For the analyses of CLEIA-PAC-final values, 13 samples from seven non-PA patients (EHT 3, non-functioning adrenal tumor without EHT 2, SCS 1, and SAI 1) and three samples from three PA patients, which included the above two samples with RIA-PAC values being less than the lower LoQ, were excluded, because their CLEIA-PAC-final values were less than the values of lower LoQ. The determination of the lower LoQ of CLEIA-PAC is described later in the part of “Relation between Accuraseed^®^ Aldosterone kit-based and SPAC^®^-S Aldosterone kit-based plasma aldosterone concentrations”. The other 221 samples from 33 non-PA and 40 PA patients were used to analyze the relation between RIA-PAC and CLEIA-PAC-final values (Table 1). For the analyses of ARR values, 102 samples, for which all of PRA, RIA-PAC, CLEIA-ARC, and CLEIA-PAC-final values were within LoQs, were used (S2 Table).

### Relation between Accuraseed^®^ renin kit-based plasma active renin concentrations and RIA-based plasma renin activities

**Fig 2:**
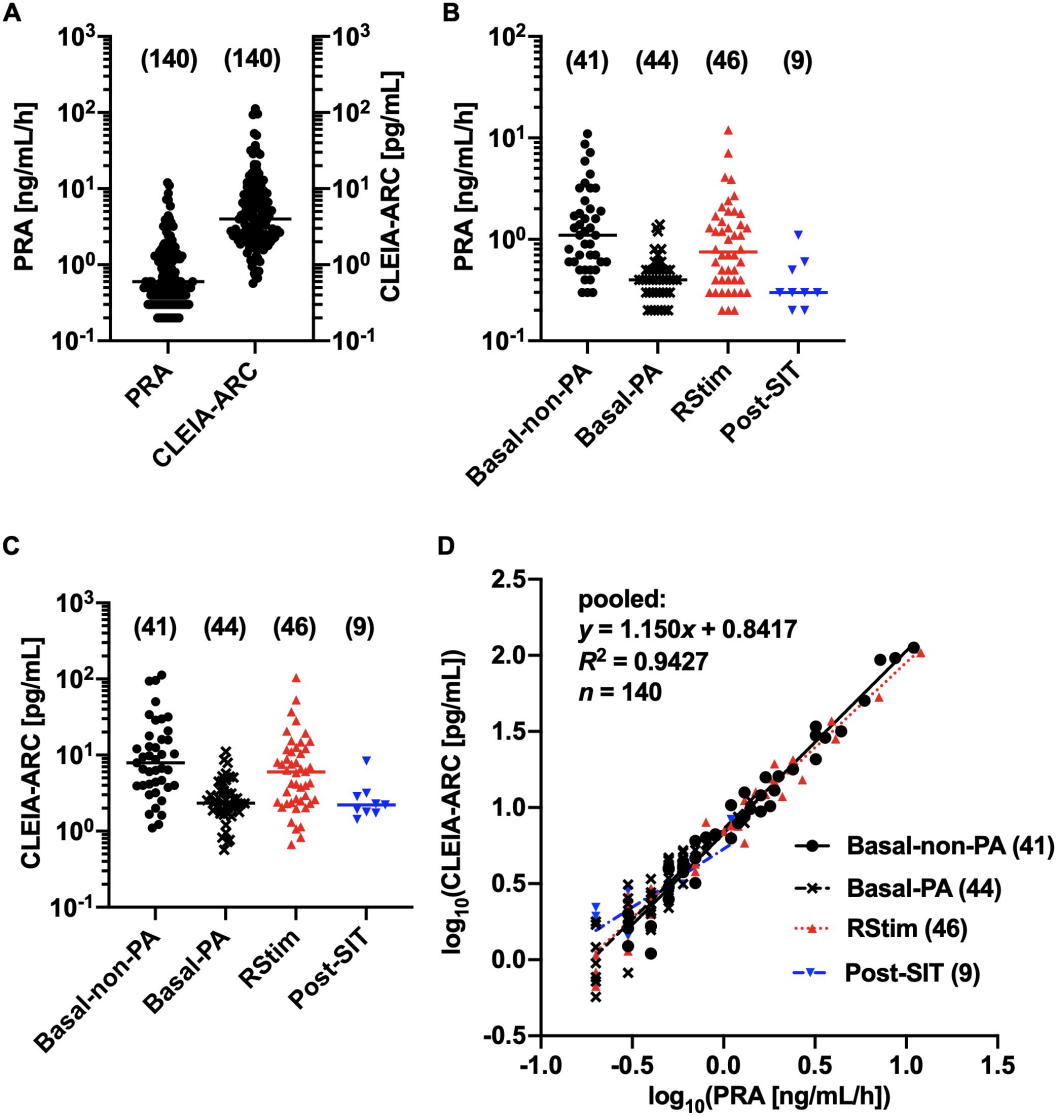
Distributions of PRA and CLEIA-ARC values and relations between them. (A) The distributions of PRA and CLEIA-ARC values of all eligible samples. (B) The distributions of PRA values of the Basal-non-PA, Basal-PA, renin stimulation test (RStim), and Post-SIT groups. (C) The distributions of CLEIA-ARC values of the above four groups. (D) The linear regression analysis between the log-transformed values of CLEIA-ARC and PRA. The equation of linear regression line by the pooled analysis is shown. The numbers of analyzed samples are shown in parentheses. Medians are indicated by horizontal bars in panels A~C.

The distributions of RIA-based plasma renin activity (PRA) and Accuraseed^®^ Renin kit-based plasma active renin concentration (CLEIA-ARC) values were not normal or log-normal, where all eligible samples were analyzed together (Fig 2A, Section A of S3 Table). However, where samples were divided into four groups, the Basal group of non-PA samples (Basal-non-PA), Basal group of PA samples (Basal-PA), and RStim and Post-SIT groups, they followed a log-normal distribution except for CLEIA-ARC values of Post-SIT group, for which the probability of log-normal distribution was much higher than that of normal distribution (Fig 2B and 2C, Section A of S3 Table). The linear regression analysis of log-transformed data gave us following regression equations with high coefficients of determination (*R*^2^), where *x* = log_10_(PRA [ng/mL/h]) and *y* = log_10_(CLEIA-ARC [pg/mL]): Basal-non-PA, *y* = 1.199*x* + 0.8297; Basal-PA, *y* = 1.191*x* + 0.8593; RStim, *y* = 1.120*x* + 0.8339; Post-SIT, *y* = 0.7654*x* + 0.7276 (Section B of S3 Table). There were no significant differences in the slopes of regression equations among the four groups, and the pooled analysis gave us the following regression equation: *y* = 1.150*x* + 0.8417 (Fig 2D, Section B of S3 Table). From this equation, the CLEIA-ARC value corresponding to 2 ng/mL/h of PRA, the cutoff value of FUP to diagnose PA in JES’s PA guidelines [9], was calculated as 15.4 pg/mL. The above equation could be transformed to the equation converting CLEIA-ARC (*x* [pg/mL]) to PRA (*y* [ng/ml/h]): $y=10^{(0.8700\times {\text{log}}_{10}x\;-\;0.7319)}$.

To compare our results with those of the previous report [15], we performed Spearman’s rank correlation and linear regression analyses between the untransformed values of CLEIA-ARC and PRA. CLEIA-ARC values showed a high-degree positive correlation with PRA values, but the slope of regression appeared to be different between low and high-value ranges (S1 Fig). The segmented linear regression analysis gave us two regression equations, where *x* = PRA [ng/mL/h] and *y* = CLEIA-ARC [pg/mL]: for *x* <2.58, *y* = 7.849*x* − 0.580, 95% CI of the slope 6.941 to ∞ (Panel A of S1 Fig); for *x* >2.58, *y* = 10.48*x* − 7.368, 95% CI of the slope 9.904 to 11.25 (Panel B of S1 Fig). The CLEIA-ARC value corresponding to 2 ng/mL/h of PRA was calculated as 15.1 pg/mL from the former equation. The limited number of post-FUP samples from non-PA patients (Table 1) prevented us from determining the optimal CLEIA-ARC cutoff for FUP by the ROC analysis.

### Relation between Accuraseed^®^ Aldosterone kit-based and SPAC^®^-S Aldosterone kit-based plasma aldosterone concentrations

#### Analyses of untransformed values and the determination of lower limit of quantitation of Accuraseed^®^ Aldosterone kit

**Fig 3:**
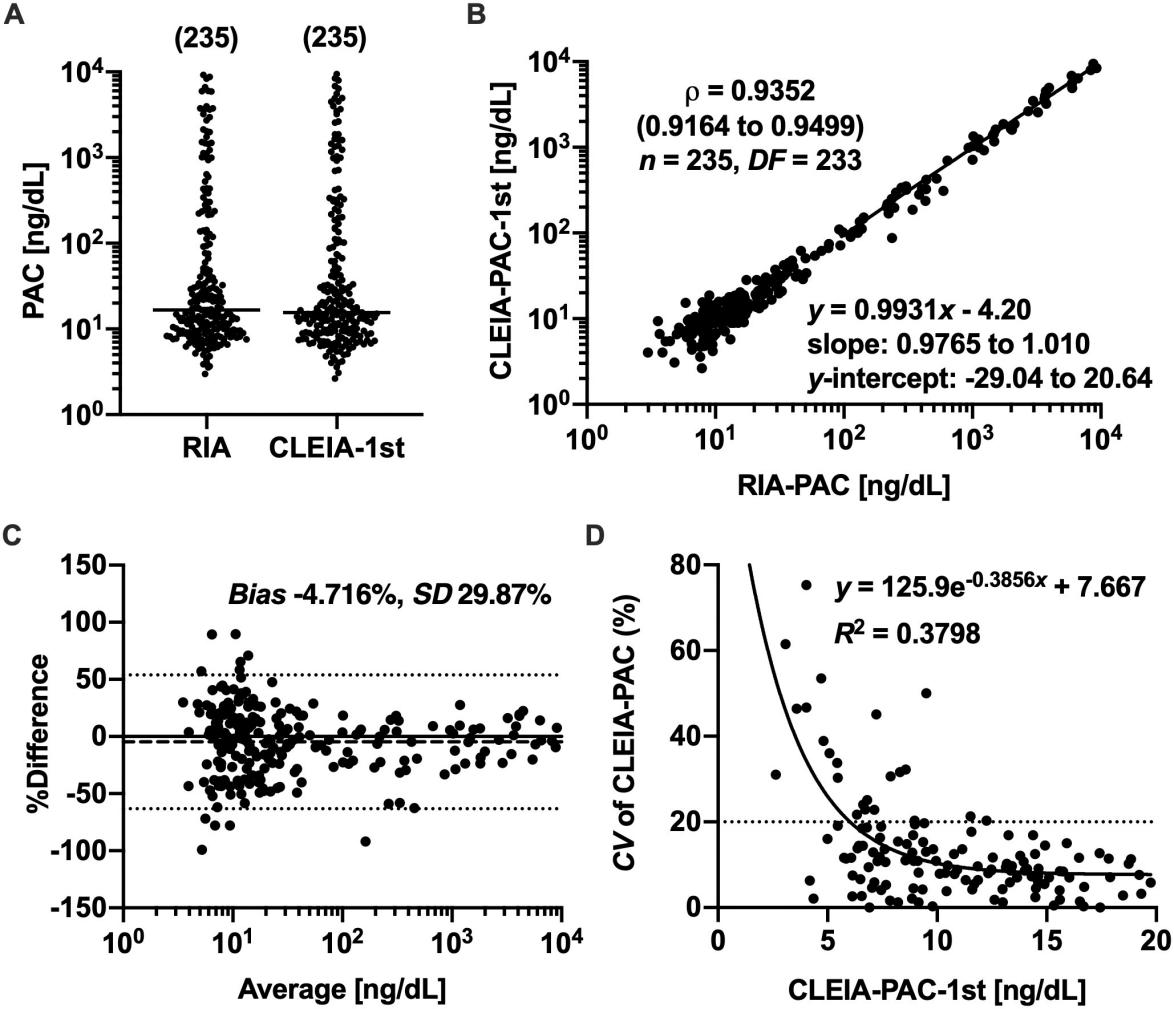
Comparisons between CLEIA-PAC-1st and RIA-PAC values. (A) The distributions of RIA-PAC and CLEIA-PAC values in the first assays (CLEIA-PAC-1st). Medians are indicated by horizontal bars and sample numbers are shown in parentheses. (B) Spearman’s rank correlation and linear regression analyses between CLEIA-PAC-1st and RIA-PAC values. Spearman’s correlation coefficient (*ρ*) is shown with the 95% CI in a parenthesis and the degree of freedom (*DF*). The equation of linear regression line is also shown with the 95% CIs of slope and *y*-intercept. (C) The Bland-Altman plot between CLEIA-PAC-1st and RIA-PAC values. The percent values of (CLEIA-PAC-1st—RIA-PAC)/(the average of CLEIA-PAC-1st and RIA-PAC) (%Difference) are plotted against the averages of CLEIA-PAC-1st and RIA-PAC values, and the bias and 95% limits of agreement are indicated with dashed and dotted lines, respectively. The bias and standard deviation (*SD*) of %Difference are also shown. (D) The relation between the coefficient of variation (*CV*) of CLEIA-PAC values and CLEIA-PAC-1st values. The *CV* of 20% is indicated by a dotted line.

The values of SPAC^®^-S Aldosterone kit-based RIA-PAC and Accuraseed^®^ Aldosterone kit-based PAC in the first assays (CLEIA-PAC-1st) did not follow a normal distribution, where all samples were analyzed together (Fig 3A, Section A of S4 Table). Spearman’s rank correlation analysis revealed a high-degree positive correlation between them (Fig 3B), but the Bland-Altman plot indicated that the agreement between them was poor because the variability of %Difference, the percent value of (CLEIA-PAC-1st—RIA-PAC)/(the average of CLEIA-PAC-1st and RIA-PAC), was high where PAC values were <20 ng/dL (Fig 3C). In order to determine if the increase of %Difference variability in the low-concentration range was caused by the poor precision of Accuraseed^®^ Aldosterone kit, we re-assayed 137 samples, of which CLEIA-PAC-1st values were <20 ng/dL. If the difference between the first and second values was greater than 20% of the first value, we performed the third assay. If the difference between the second and third values was greater than 20% of the second value, we performed the fourth assay. Then, the CLEIA-PAC-final value was determined to be the average of the nearest two measured values of each sample. Where the CLEIA-PAC-1st value was ≥20 ng/dL, it was used as the CLEIA-PAC-final value. In the re-assayed samples, the *CV* of CLEIA-PAC values for each sample showed a one-phase exponential decay as the CLEIA-PAC-1st value increases, and it was estimated to be >20% where CLEIA-PAC-1st values were <6 ng/dL (Fig 3D, Section D of S4 Table); the lower LoQ of CLEIA-PAC was determined to be 6 ng/dL. Actually, the *CV* values of Accuraseed^®^ Aldosterone kit-based aldosterone concentrations for aldosterone solutions prepared in the steroid-deprived serum were <20% where aldosterone concentrations were ≥6 ng/dL; the *CV* values for 6, 9, 12, 15, 40, and 80-ng/dL aldosterone were 12.1%, 7.9%, 5.2%, 3.9%, 2.0%, and 1.3%, respectively (Section A of S5 Table). The *CV* values of CLEIA-PAC for patient samples with CLEIA-PAC-1st values being ≥20 ng/dL were 0.18~3.8% (Section B of S5 Table). We, therefore, excluded 14 samples with CLEIA-PAC-final values <6 ng/dL from the 235 samples shown in Fig 3B and used the remaining 221 samples for further analyses. Although CLEIA-PAC-final values were not normally distributed (Panel A of S2 Fig, Section A of S6 Table), we analyzed the linear relation of CLEIA-PAC-final values (*y* [ng/dL]) to RIA-PAC values (*x* [ng/dL]) to compare our results with those in the previous report [15]. We obtained the following regression equation: *y* = 0.9931*x* − 3.98 (Panel B of S2 Fig, Section B of S6 Table). The correlation coefficient and agreement between CLEIA-PAC-final and RIA-PAC values tended to be better than those between CLEIA-PAC-1st and RIA-PAC values (Fig 3B and 3C, Panels B and C of S2 Fig, S4 and S6 Tables). Hereafter, we described CLEIA-PAC-final values as CLEIA-PAC values, unless otherwise stated.

#### Analyses of log-transformed values

**Fig 4:**
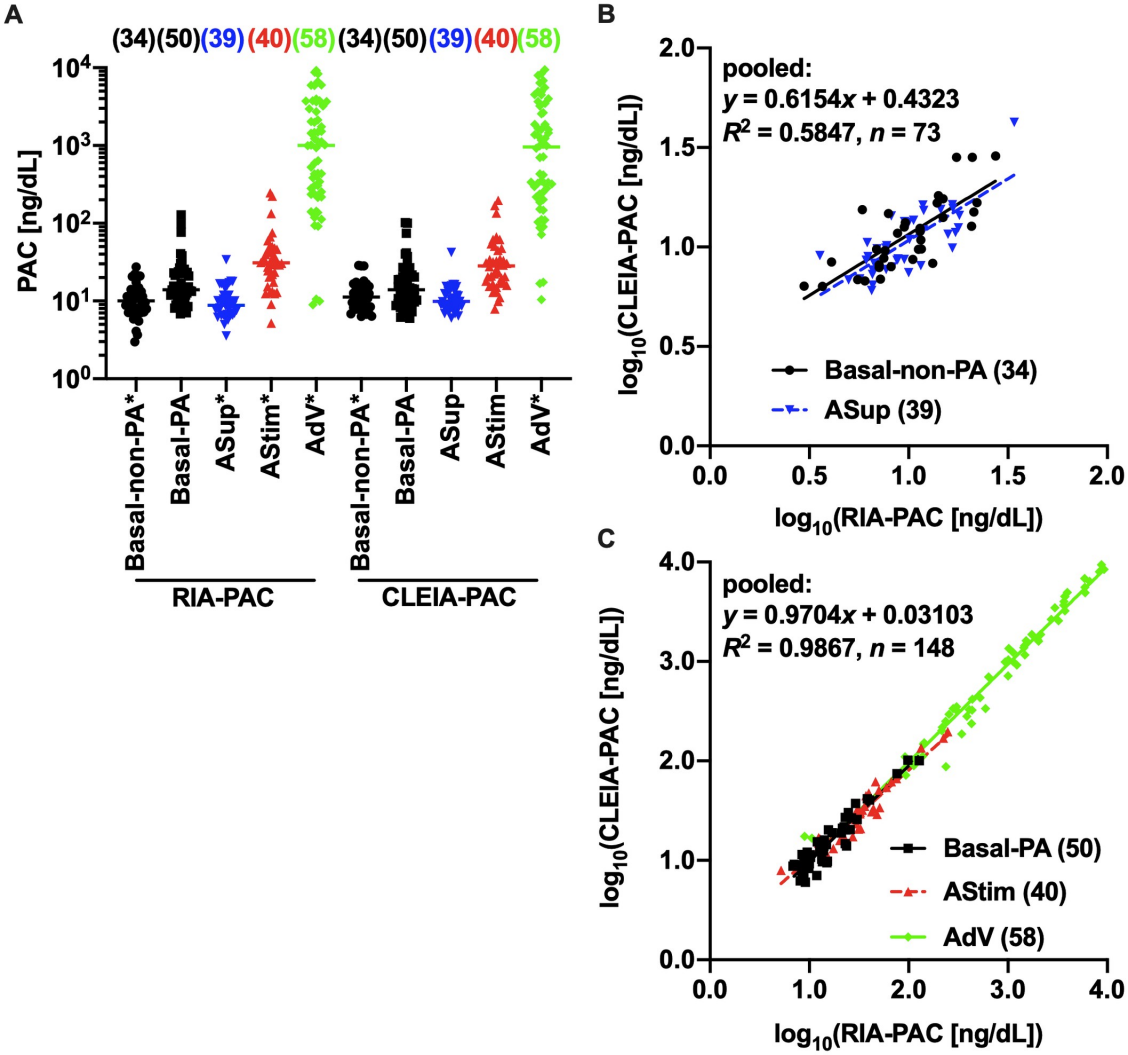
Distributions of RIA-PAC and CLEIA-PAC (CLEIA-PAC-final) values and relations between them in different sampling conditions. (A) The distributions of RIA-PAC and CLEIA-PAC values in the Basal-non-PA, Basal-PA, aldosterone suppression test (ASup), aldosterone stimulation test (AStim), and adrenal vein (AdV) groups. Groups, of which data are log-normally distributed, are indicated with *. For the other groups, the likelihood of log-normal distribution was much higher than that of normal distribution. Linear regression analyses between the log-transformed values of CLEIA-PAC and RIA-PAC in the complex A (the Basal-non-PA and ASup groups) (B) and in the complex B (the other groups) (C) are shown. The numbers of analyzed samples are shown in parentheses.

CLEIA-PAC and RIA-PAC values appeared to be log-normally distributed in each group, where samples were divided into five groups: Basal-non-PA, Basal-PA, ASup, AStim, and AdV (Fig 4A, Section A of S7 Table). RIA-PAC values in the Basal-PA group and CLEIA-PAC values in the Basal-PA, ASup, and AStim groups did not pass the normality test after log-transformations, but the likelihood of log-normal distribution was much higher than that of normal distribution (Section A of S7 Table). We, therefore, performed linear regression analyses between log-transformed values. It gave us following regression equations, where *x* = log_10_(RIA-PAC [ng/dL]) and *y* = log_10_(CLEIA-PAC [ng/dL]): Basal-non-PA, *y* = 0.6137*x* + 0.4498; Basal-PA, *y* = 0.9619*x* + 0.02614; ASup, *y* = 0.6174*x* + 0.4164; AStim, *y* = 0.8876*x* + 0.1322; AdV, *y* = 0.9839*x* + 0.02059 (Section B of S7 Table). Although slopes were significantly different among the five groups, they were not significantly different within the complex A (the Basal-non-PA and ASup groups) and the complex B (the Basal-PA, AStim, and AdV groups) (Section B of S7 Table). Pooled regression analyses in complexes A and B provided regression equations A (*y* = 0.6154*x* + 0.4323) and B (*y* = 0.9704*x* + 0.03103), respectively (Fig 4B and 4C, Section B of S7 Table). CLEIA-PAC values corresponding to 12 and 1,400 ng/dL of RIA-PAC, which are cutoff values for PA screening with peripheral vein samples in the basal condition and aldosterone hypersecretion in AdV samples in JES’s PA guidelines [9], were calculated from the equation B as 12 and 1,213 ng/dL, respectively. CLEIA-PAC values corresponding to 12 and 6 ng/dL of RIA-PAC, which are cutoff values for CCT and SIT in JES’s PA guidelines [9], were calculated from the equation A as 12 and 8.2 ng/dL, respectively. Because the limited number of post-CCT and post-SIT samples and no AdV samples were obtained from non-PA patients (Table 1), we gave up checking the above-mentioned cutoffs by ROC analyses. The equations A and B could be transformed to the equations a and b converting CLEIA-PAC (*x* [ng/dL]) to RIA-PAC (*y* [ng/dL]): $y=10^{(1.625\times {\text{log}}_{10}x\;-\;0.7025)}$ (the equation a for the complex A) and $y=10^{(1.031\times {\text{log}}_{10}x\;-\;0.03198)}$ (the equation b for the complex B).

### Relation between Accuraseed^®^ immunoanalyzer-based and RIA-based aldosterone-to-renin ratios

**Fig 5:**
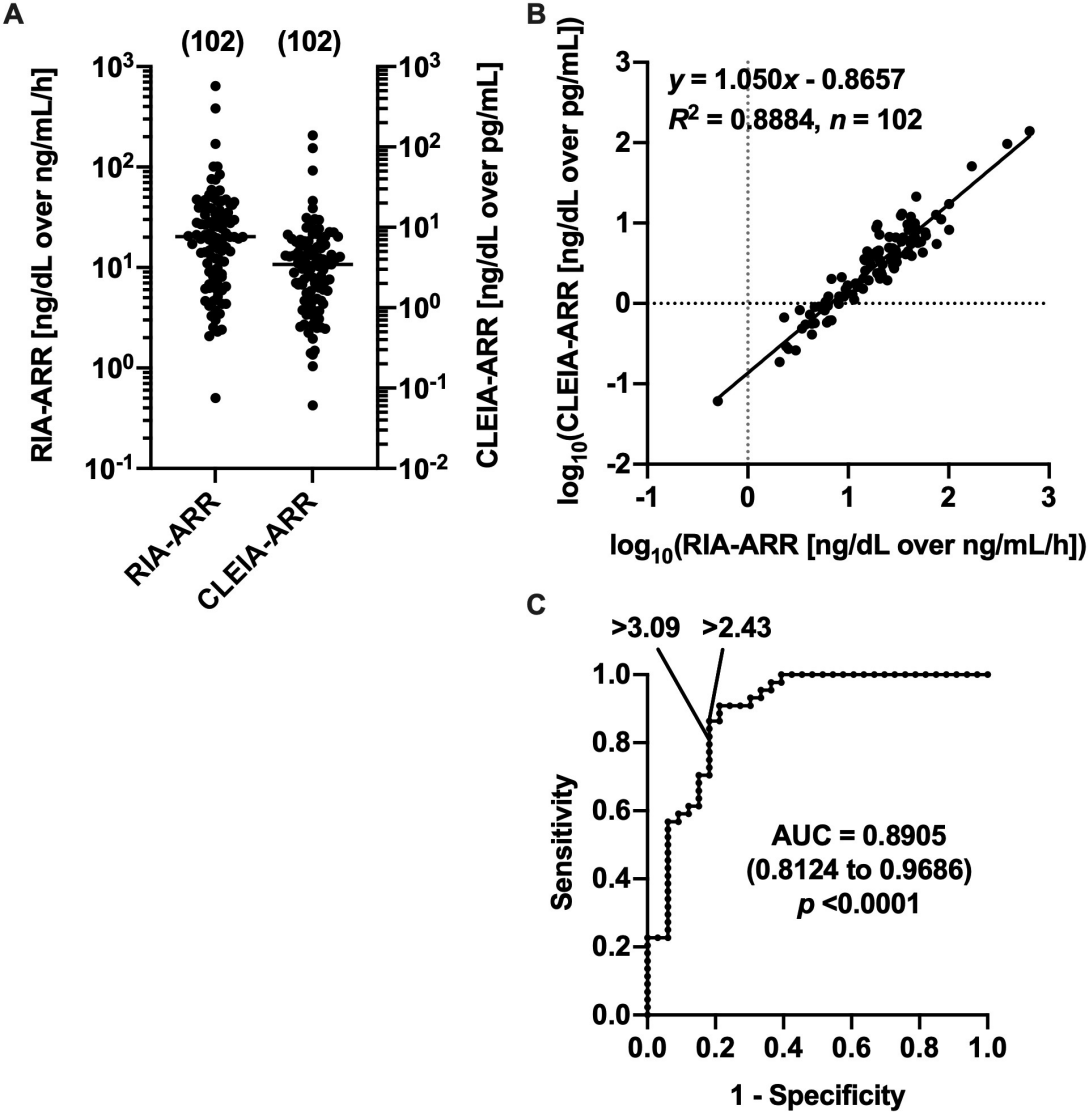
Analyses of RIA-ARR and CLEIA-ARR values. In the samples of Basal group and samples at 60 min after the loading of captopril challenge test (CCT60), the distributions of RIA-ARR and CLEIA-ARR values (A) and the linear regression analysis between their log-transformed values (B) are shown. In panel A, the numbers of samples analyzed are shown in parentheses and medians are indicated by horizontal bars. (C) The ROC analysis to determine the cutoff of basal CLEIA-ARR to detect PA patients. For the ROC analysis, only the samples of Basal group are used; numbers of non-PA and PA samples are 33 and 44, respectively. The area under the curve (AUC) of ROC curve is shown with the 95% CI in a parenthesis and *p* value. The cutoff maximizing the Youden index (>2.43 ng/dL over pg/mL) and that corresponding to RIA-ARR of 20 ng/dL over ng/mL/h (>3.09 ng/dL over pg/mL) are indicated.

We investigated the relation between Accuraseed^®^ immunoanalyzer-based aldosterone-to-renin ratio (CLEIA-ARR) and RIA-ARR values in the samples of Basal group and CCT60 samples (S2 Table). RIA-ARR and CLEIA-ARR values showed log-normal distributions (Fig 5A, Section A of S8 Table), and log-transformed CLEIA-ARR values showed a high-degree positive correlation with log-transformed RIA-ARR values (*r* = 0.9778, 95% CI 0.9673 to 0.9850, *p* <0.0001, *n* = 102). Where *x* = log_10_(RIA-ARR [ng/dL over ng/mL/h]) and *y* = log_10_(CLEIA-ARR [ng/dL over pg/mL]), the regression equation was *y* = 1.050*x* − 0.8657 (Fig 5B, Section B of S8 Table). Where samples were divided into the Basal group and CCT60 samples, RIA-ARR and CLEIA-ARR values in both groups showed log-normal distributions (Section A of S9 Table). If regression analyses were performed separately in the Basal group and CCT60 samples, the slopes of regression equations were not different between the two groups (Section B of S9 Table). From the above equation, the CLEIA-ARR value corresponding to 20 ng/dL over ng/mL/h of RIA-ARR, which is the cutoff value for PA screening and CCT to diagnose PA in JES’s PA guidelines [9], was calculated as 3.09 ng/dL over pg/mL. If CLEIA-ARR >3.09 ng/dL over pg/mL was used for PA screening, the sensitivity and specificity were 81.8% and 81.8%, respectively, while the optimal cutoff determined by the ROC analysis was CLEIA-ARR >2.43 ng/dL over pg/mL detecting PA with the sensitivity of 86.4% and the specificity of 81.8% (Fig 5C, S10 Table). We did not perform the ROC analysis to determine the optimal cutoff of post-CCT CLEIA-ARR values to diagnose PA because the number of post-CCT samples from non-PA patients was limited (Table 1). The above equation could be transformed to the equation converting RIA-ARR (*x* [ng/dL over ng/mL/h]) to CLEIA-ARR (*y* [ng/dL over pg/mL]): $y=10^{(0.9524\times {\text{log}}_{10}x\;+\;0.8245)}$.

## Discussion

### Prevalence of primary aldosteronism among participants

Although the prevalence of primary aldosteronism (PA) among patients with hypertension was reported to be 5~10% [9, 10], about half of participants were diagnosed with PA in the present study. Because patients highly suspected of PA had been preferentially referred to our center, the prevalence of PA among our patients should be much higher than that among patients with hypertension in general practice. Actually, we previously reported that 128 (90.1%) of 142 patients, who received PA confirmatory tests for the scrutiny of PA in our center from April 2012 to December 2017, were PA patients [28]. On the other hand, patients whose PAC or PRA values had been measured in the scrutiny of adrenal incidentaloma, adrenal insufficiency, and renovascular hypertension were also included in the present study (Fig 1). Most of them did not fulfill the screening criteria of PA and were diagnosed with non-PA without PA confirmatory tests being performed. Taken together, the prevalence of PA among participants in the present study is consistent with the design of the present study.

### Relation between Accuraseed^®^ Renin kit-based plasma active renin concentrations and RIA-based plasma renin activities

Since the values of RIA-based PRA and Accuraseed^®^ Renin kit -based CLEIA-ARC appeared to be log-normally distributed in each sample group (S3 Table), linear regression analyses should be performed between log-transformed values in each group. Log-transformed CLEIA-ARC values showed a linear relation to log-transformed PRA values with high *R*^2^. There were no significant differences in the slopes of linear regression equations among sample groups, indicating that sampling conditions did not affect the relation (Fig 2D, Section B of S3 Table). If we performed the linear regression analysis between untransformed values as in the previous study [15], we obtained the following regression equation, where *x* = PRA (ng/mL/h) and *y* = CLEIA-ARC (pg/mL): *y* = 9.626*x* − 1.956 (S11 Table). The regression equation was different from that reported previously: *y* = 4.082*x* + 0.549 [15]. While PRA values were less than 3 ng/mL/h in the previous report [15], they were up to 12 ng/mL/h in the present study (Fig 2); the relation might be different in the high-level range (PRA >3 ng/mL/h). We, therefore, performed the segmented linear regression analysis and found that the slope of the linear regression line became steeper at 2.58 ng/mL/h of PRA (S1 Fig). In the previous study, PRA values were measured by the Renin RIABEAD^®^ kit [15], which was different from the kit used in the present study. This difference should be the major cause of the difference in results. The equation obtained in the present study should be used to convert PRA values measured by the PRA-FR^®^ RIA kit to CLEIA-ARC values. From either equation of the segmented linear regression between untransformed values and linear regression between log-transformed values, the CLEIA-ARC value corresponding to 2 ng/mL/h of PRA, the cutoff value of FUP to diagnose PA in JES’s PA guidelines [9], was calculated as about 15 pg/mL. It would be appropriate to set the cutoff of post-FUP CLEIA-ARC to diagnose PA as <15 pg/mL.

### Relation between Accuraseed^®^ Aldosterone kit-based and SPAC^®^-S Aldosterone kit-based plasma aldosterone concentrations

The Bland-Altman plot indicated that the bias between the first values of Accuraseed^®^ Aldosterone kit-based CLEIA-PAC (CLEIA-PAC-1st) and SPAC^®^-S Aldosterone kit-based RIA-PAC values was small but that the variability of %Difference was high in the low-concentration range (Fig 3C). Because the *CV* of CLEIA-PAC increased as the CLEIA-PAC-1st value decreased (Fig 3D), the high variability of %Difference was suggested to be caused by the high variability of Accuraseed^®^ Aldosterone kit-based CLEIA-PAC values, which depended not on systematic errors such as differences in the cross-reactivities of immunoassays but on the instability of measured CLEIA-PAC values in the low-concentration range. The determination of CLEIA-PAC-final values by multiple assays reduced the risk of bringing measurement errors into the analyses. In the present study, the lower LoQ of CLEIA-PAC was determined to be 6 ng/dL (Fig 3D), which was similar to that in the previous study (5 ng/dL) [15]. On the other hand, where CLEIA-PAC-1st values were ≥15 ng/dL, CLEIA-PAC values were well reliable because the *CV* values were <5% (S5 Table). Where CLEIA-PAC-1st values were >10 ng/dL, re-assays would not be necessary because the *CV* values of CLEIA-PAC were estimated and confirmed to be <10% (Fig 3D, Section A of S5 Table). The intra-assay precision of CLEIA-PAC in the present study was similar to that reported previously: *CV* values of CLEIA-PAC for plasma samples with 18.18, 45.73, and 113.35-ng/dL PAC were reported to be 5.8%, 2.7%, and 1.4%, respectively [15]. The linear regression equation between CLEIA-PAC (*y* [ng/dL]) and RIA-PAC (*x* [ng/dL]) values was previously reported as *y* = 1.042*x* + 1.478 [15]; the slope of regression equation was different from that in the present study (Panel B of S2 Fig, Section B of S6 Table). The inclusion of non-basal samples in the present study might result in this difference.

Since the values of RIA-PAC and CLEIA-PAC appeared to be log-normally distributed in each sample group (Section A of S7 Table), linear regression analyses should be performed between log-transformed values in each group. The Basal-non-PA and ASup groups could be combined into a complex (A), and the other groups could be combined into the other complex (B). The regression equations were different between the two complexes: the equations A and B for the complexes A and B, respectively (Fig 4B and 4C). Although PAC values were obtained by measuring diluted samples for most AdV samples, the AdV group could share the same regression equation with the Basal-PA and AStim groups, for which PAC values were measured without dilutions; dilution procedures did not affect the regression equation (Fig 4C, Section B of S7 Table). The cutoffs of basal CLEIA-PAC for PA screening and AdV CLEIA-PAC for aldosterone hypersecretion could be calculated from the regression equation B with high *R*^2^ (Fig 4C). CLEIA-PAC cutoffs for SIT and CCT to diagnose PA could be calculated from the regression equation A, but *R*^2^ was not so good (Fig 4B). This might be due to the relatively poor precisions of not only Accuraseed^®^ Aldosterone kit-based CLEIA-PAC (Section A of S5 Table) but also RIA-PAC [22] in the range of PAC <15 ng/dL.

The assay kit, by which clinical judgement values have been established, should be called the standard assay kit. Currently, the SPAC^®^-S Aldosterone kit is the standard assay kit for PAC in Japan. As described in the previous report [15], the present study revealed that Accuraseed^®^ Aldosterone kit-based CLEIA-PAC values were different from SPAC^®^-S Aldosterone kit-based RIA-PAC values although a high-degree positive correlation was observed between them. If we use the Accuraseed^®^ Aldosterone kit to measure PAC values and the clinical judgement values established by the SPAC^®^-S Aldosterone kit-based RIA-PAC to screen for and diagnose PA, we should convert Accuraseed^®^ Aldosterone kit-based CLEIA-PAC values to SPAC^®^-S Aldosterone kit-based RIA-PAC values. The present study provided regression equations a and b for this conversion. The former was for post-CCT and post-SIT samples, and the Basal group of non-PA samples (the complex A), and the latter was for post-FUP, post-ACTH, and AdV samples, and the Basal group of PA samples (the complex B). However, we could not decide which conversion equation should be used for basal samples until the diagnosis of PA or non-PA was done. The difference in relations between Accuraseed^®^ Aldosterone kit-based CLEIA-PAC values and SPAC^®^-S Aldosterone kit-based RIA-PAC values might depend not on the diagnosis but on PAC levels; regression equations a and b might be those for low and high PAC ranges. The cutoff between the ranges appeared to be 15 ng/dL of CLEIA-PAC because the 75th percentile of CLEIA-PAC in the complex A and the 25th percentile of CLEIA-PAC in the complex B were around this value (S12 Table).

### Relation between Accuraseed^®^ immunoanalyzer-based and RIA-based aldosterone-to-renin ratios

The linear regression analysis between the log-transformed values of Accuraseed^®^ immunoanalyzer-based CLEIA-ARR and RIA-ARR indicated that the CLEIA-ARR value corresponding to the RIA-ARR cutoff used both to screen for PA and diagnose PA with CCT in JES’s PA guidelines (>20 ng/dL over ng/mL/h) [9] was >3.09 ng/dL over pg/mL. On the other hand, the optimal CLEIA-ARR cutoff for PA screening was determined to be >2.43 ng/dL over pg/mL by the ROC analysis (Fig 5C, S10 Table). Because the latter provided higher sensitivity than that of the former with the specificity being the same, the latter was better as the cutoff for PA screening. However, the optimal cutoff value of basal CLEIA-ARR to detect PA patients was previously reported to be >6 ng/dL over pg/mL, where PA was diagnosed only by CCT [15]. Because 77% of CCT-negative patients were diagnosed with PA by SIT or FUP in our previous report [28], the cutoff value should be decreased to pick up CCT-negative PA patients. If PA had been diagnosed only by CCT in the present study, ten of 44 PA patients would have been diagnosed with non-PA and the optimal cutoff of basal CLEIA-ARR to detect PA would have increased to >3.47 ng/dL over pg/mL (Panel A of S3 Fig, S13 Table). While the prevalence of patients with unilateral PA was very high in the previous study (75 of 125 PA cases) [15], only four of 24 Basal-PA samples, for which the laterality of aldosterone hypersecretion was determined by AVS, were unilateral PA samples in the present study (Panel A of S4 Fig). CLEIA-ARR values of unilateral PA samples tended to be higher than those of bilateral PA samples (*p* = 0.1826) in the present study (Panel A of S4 Fig, S14 Table), as observed in the previous study [15]. The higher prevalence of unilateral disease in PA patients in the previous study might result in the higher optimal CLEIA-ARR cutoff for PA screening. In the present study, although the number of unilateral PA samples was small, the optimal, basal CLEIA-ARR cutoff to distinguish unilateral PA samples from non-PA samples was determined to be >5.63 ng/dL over pg/mL by the ROC analysis (Panel B of S3 Fig, S15 Table), which was almost equal to the cutoff for PA screening in the previous study [15]. In the present study, eight of 20 bilateral PA samples showed basal CLEIA-ARR values less than 6 ng/dL over pg/mL, but no unilateral PA samples did (Panel A of S4 Fig). Taken together, >2.43 ng/dL over pg/mL would be appropriate as the cutoff of basal CLEIA-ARR for PA screening not to overlook bilateral PA. By contrast, no non-PA CCT60 samples showed CLEIA-ARR values >3.09 ng/dL over pg/mL, which corresponded to >20 ng/dL over ng/mL/h of RIA-ARR (Panel B of S4 Fig). Post-CCT CLEIA-ARR >3.09 ng/dL over pg/mL would be tentatively appropriate as the cutoff of CCT to diagnose PA, but an ROC analysis with samples of large number will be needed to obtain a conclusive result. In the present study, we examined the relation between CLEIA-ARR values calculated by dividing CLEIA-PAC by CLEIA-ARC and RIA-ARR values calculated by dividing RIA-PAC by PRA. If we had first converted CLEIA-PAC and CLEIA-ARC values to RIA-PAC and PRA values, respectively, and had calculated ARR values, the values could be different from RIA-ARR values.

### Limitations

Firstly, the compositions of samples were not uniform among sampling conditions; non-PA samples were dominant in basal samples, but PA samples were dominant in CCT60, post-SIT, post-FUP, and AdV samples (Table 1). This difference could affect relations between CLEIA-based and RIA-based values, but relations with log-log transformations between CLEIA-ARC and PRA values and those between CLEIA-ARR and RIA-ARR values were not different among sampling conditions (Fig 2D, S3 and S9 Tables). By contrast, relations with log-log transformations between CLEIA-PAC and RIA-PAC values were different between two complexes, and Basal-PA and Basal-non-PA samples belonged to different complexes (Fig 4B and 4C, Section B of S7 Table). However, the Basal-non-PA group shared the same regression line with the ASup group, in which PA samples were dominant (Fig 4B, Table 1). The difference in relations appeared to depend not on the diagnosis but on PAC levels as discussed above.

Secondly, the numbers of CCT60, post-SIT, and post-FUP samples from non-PA patients in the present study were too small to accurately determine the optimal cutoffs of CLEIA-ARR, CLEIA-PAC, and CLEIA-ARC for CCT, SIT, and FUP, respectively, using ROC analyses (Table 1). Since the ROC-based optimal CLEIA-ARR cutoff value for PA screening was different from the value, which was calculated from the RIA-ARR cutoff value for PA screening by the regression equation, optimal CLEIA-based cutoff values for CCT, SIT, and FUP might be different from the calculated values. In the range of PAC <15 ng/dL, the precision of CLEIA-PAC was relatively poor (S5 Table) and the precision of RIA-PAC was also not so good [22]. Because *R*^2^ was not so good for the linear relation with log-log transformations between CLEIA-PAC and RIA-PAC values in the ASup group (Fig 4B), in which most samples showed CLEIA-PAC <15 ng/dL, we should keep the uncertainty of the calculated CLEIA-PAC cutoff values for CCT and SIT in mind.

Thirdly, the present study is a single-center study, and the numbers of samples and participants are relatively small. The results are not conclusive and multi-center-type validation studies of larger sample sizes will be needed.

## Conclusions

Where several different immunoassays are available to measure a hormone, the harmonization of the assays are necessary to use the same clinical judgment values for the hormone. However, the harmonization of assays for aldosterone and renin has not been established. The present study provided equations converting Accuraseed^®^ Aldosterone kit-based CLEIA-PAC values to SPAC^®^-S Aldosterone kit-based RIA-PAC values, and this is the first report to provide the equations for samples of loading tests and AVS. The present study also first provided equations converting Accuraseed^®^ Renin kit-based CLEIA-ARC and Accuraseed^®^ immunoanalyzer-based CLEIA-ARR values to PRA-FR^®^ RIA kit-based PRA and RIA-ARR values, respectively. Although validation studies of larger sample sizes will be needed, the present study will hopefully become priming water of harmonization of aldosterone and renin immunoassays.

## Supporting information

S1 Fig Relations between CLEIA-ARC and PRA values. The results of segmented linear regression analysis between the untransformed values of Accuraseed^®^ Renin kit-based active renin concentration (CLEIA-ARC) and radioimmunoassay-based plasma renin activity (PRA) are shown separately in the range of PRA being <2.58 ng/mL/h (A) and in the range of PRA being >2.58 ng/mL/h (B). The linear regression equation for the corresponding segment is shown in each panel. The coefficient of determination (*R*^2^), *n*, and the degree of freedom (*DF*) of the analysis including both panels are 0.9637, 140, and 135, respectively. Spearman’s correlation coefficient *ρ* is 0.9579 with the 95% confidence interval of 0.9411–0.9700 (*n* = 140, *DF* = 138).
(TIF)

S2 Fig Relations between CLEIA-PAC-final and RIA-PAC values. (A) The distribution of the final values of Accuraseed^®^ Aldosterone kit-based plasma aldosterone concentration (CLEIA-PAC-final). The median is indicated by a horizontal bar. The number of samples is shown in a parenthesis. (B) Spearman’s rank correlation and linear regression analyses between CLEIA-PAC-final and radioimmunoassay-based plasma aldosterone concentration (RIA-PAC) values. Spearman’s correlation coefficient *ρ* is shown with the 95% confidence interval (CI) in a parenthesis and the degree of freedom (*DF*). The equation of linear regression line is also shown with the 95% CIs of slope and *y*-intercept. (C) The Bland-Altman plot between CLEIA-PAC-final and RIA-PAC values. The percent values of (CLEIA-PAC-final—RIA-PAC)/(the average of CLEIA-PAC-final and RIA-PAC) (%Difference) are plotted against the averages of CLEIA-PAC-final and RIA-PAC values, and the 95% limits of agreement are indicated with dotted lines.
(TIF)

S3 Fig Detecting patients with captopril challenge test-based or unilateral primary aldosteronism by CLEIA-ARR. (A) The receiver operating characteristic (ROC) analysis of Accuraseed^®^ immunoanalyzer-based aldosterone-to-renin ratio (CLEIA-ARR) to detect patients with primary aldosteronism (PA) diagnosed only by the captopril challenge test (CCT). Numbers of non-PA and PA samples are 43 and 34, respectively. (B) The ROC analysis of CLEIA-ARR to distinguish unilateral PA patients from non-PA patients. In the analysis shown in this panel, PA was diagnosed by not only CCT. Numbers of non-PA and unilateral PA samples are 34 and 4, respectively. Areas under the curves (AUCs) are shown with 95% confidence intervals in parentheses and *p* values. Optimal cutoffs are also shown in ng/dL over pg/mL. The data of Basal group, which consists of basal, ambulatory, and before loading samples, and inferior vena cava samples before the adrenocorticotropic hormone loading in the adrenal venous sampling, are used.
(TIF)

S4 Fig Distributions of CLEIA-ARR values divided by diagnoses. (A) The distributions of Accuraseed^®^ immunoanalyzer-based aldosterone-to-renin ratio (CLEIA-ARR) values in the samples of Basal group, for which AVS was successfully performed, divided by the laterality of PA. (B) The distributions of CLEIA-ARR values in the samples of non-PA and PA patients at 60 min after the loading of captopril challenge test (CCT60). In panels A and B, dotted lines indicate 6 and 3.09 ng/dL over pg/mL of CLEIA-ARR, respectively. Medians are indicated by horizontal bars. Sample numbers are shown in parentheses.
(TIF)

S1 Table Cross-reactivities of assay kits used in the present study. (DOCX)

S2 Table Numbers and classifications of samples for aldosterone-to-renin ratio analyses. (DOCX)

S3 Table Distributions of PRA and CLEIA-ARC values and relations between them. (DOCX)

S4 Table Distributions of RIA-PAC and CLEIA-PAC-1st values and relations between them. (DOCX)

S5 Table Intra-assay precision of Accuraseed^®^ Aldosterone kit. (DOCX)

S6 Table Relations between CLEIA-PAC-final and RIA-PAC value. (DOCX)

S7 Table Distributions of RIA-PAC and CLEIA-PAC values and relations between them. (DOCX)

S8 Table Distributions of RIA-ARR and CLEIA-ARR values and the relation between them. (DOCX)

S9 Table Distributions of RIA-ARR and CLEIA-ARR values and their relations in two sample groups. (DOCX)

S10 Table Detecting primary aldosteronism with Accuraseed^®^ immunoanalyzer-based aldosterone-to-renin ratios. (XLSX)

S11 Table The relation between CLEIA-ARC and PRA values. (DOCX)

S12 Table Descriptive statistics of CLEIA-PAC values. (DOCX)

S13 Table Detecting primary aldosteronism diagnosed only by the captopril challenge test with CLEIA-ARR values. (XLSX)

S14 Table Difference of CLEIA-ARR values between lateralities of primary aldosteronism. (DOCX)

S15 Table Detecting unilateral primary aldosteronism with Accuraseed^®^ immunoanalyzer-based aldosterone-to-renin ratios. (XLSX)
