# Supplementary figures and images for: Comparisons of plasma aldosterone and renin data between an automated chemiluminescent immunoanalyzer and conventional radioimmunoassays in the screening and diagnosis of primary aldosteronism
Source: PLoS One. 2021 Jul 9;16(7):e0253807. doi: 10.1371/journal.pone.0253807 (PMC8270132; doi:10.1371/journal.pone.0253807)

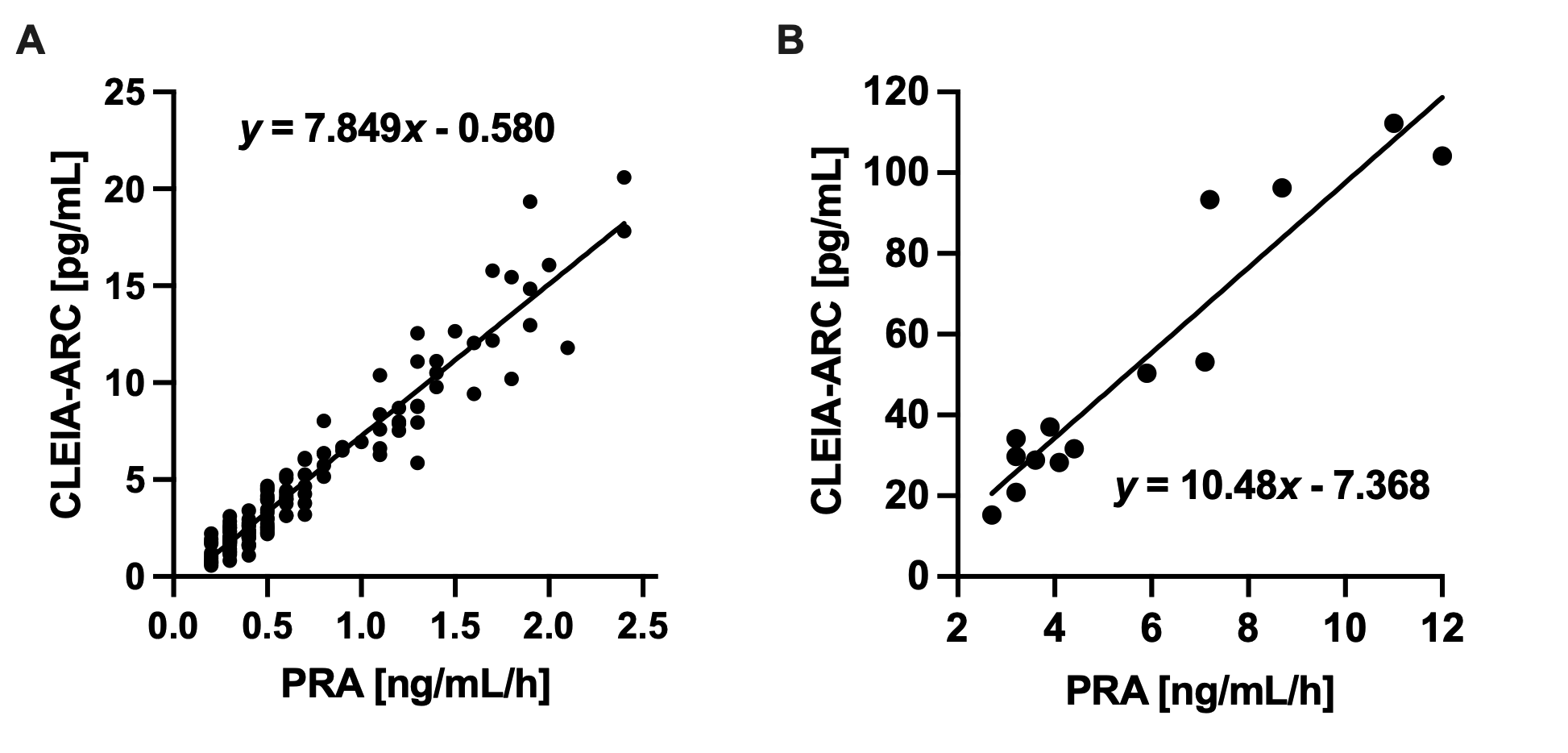

Supplement: S1 Fig — The results of segmented linear regression analysis between the untransformed values of Accuraseed® Renin kit-based active renin concentration (CLEIA-ARC) and radioimmunoassay-based plasma renin activity (PRA) are shown separately in the range of PRA being <2.58 ng/mL/h (A) and in the range of PRA being >2.58 ng/mL/h (B). The linear regression equation for the corresponding segment is shown in each panel. The coefficient of determination (R2), n, and the degree of freedom (DF) of the analysis including both panels are 0.9637, 140, and 135, respectively. Spearman’s correlation coefficient ρ is 0.9579 with the 95% confidence interval of 0.9411–0.9700 (n = 140, DF = 138). (TIF) [file pone.0253807.s001.tif]

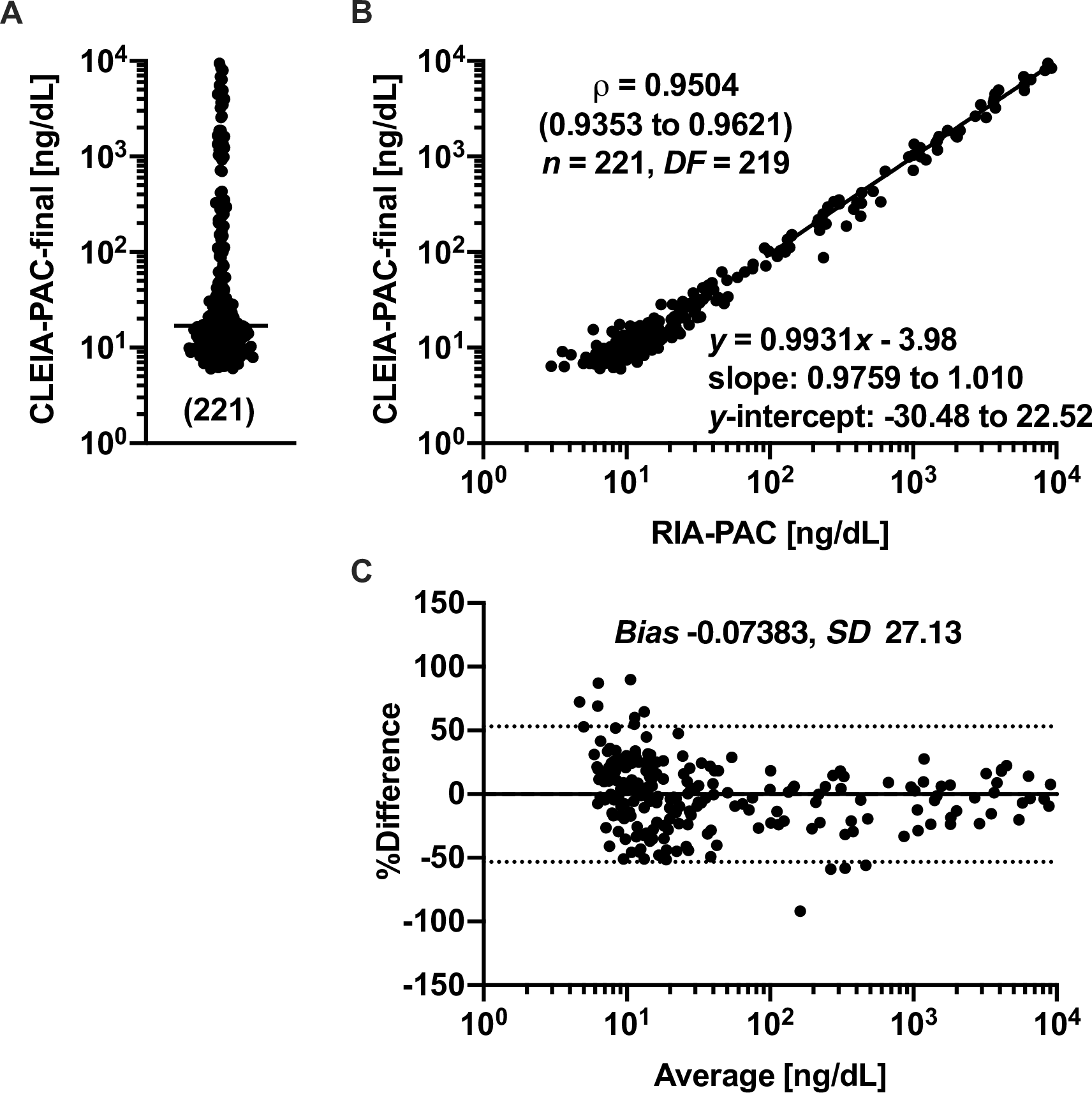

Supplement: S2 Fig — (A) The distribution of the final values of Accuraseed® Aldosterone kit-based plasma aldosterone concentration (CLEIA-PAC-final). The median is indicated by a horizontal bar. The number of samples is shown in a parenthesis. (B) Spearman’s rank correlation and linear regression analyses between CLEIA-PAC-final and radioimmunoassay-based plasma aldosterone concentration (RIA-PAC) values. Spearman’s correlation coefficient ρ is shown with the 95% confidence interval (CI) in a parenthesis and the degree of freedom (DF). The equation of linear regression line is also shown with the 95% CIs of slope and y-intercept. (C) The Bland-Altman plot between CLEIA-PAC-final and RIA-PAC values. The percent values of (CLEIA-PAC-final—RIA-PAC)/(the average of CLEIA-PAC-final and RIA-PAC) (%Difference) are plotted against the averages of CLEIA-PAC-final and RIA-PAC values, and the 95% limits of agreement are indicated with dotted lines. (TIF) [file pone.0253807.s002.tif]

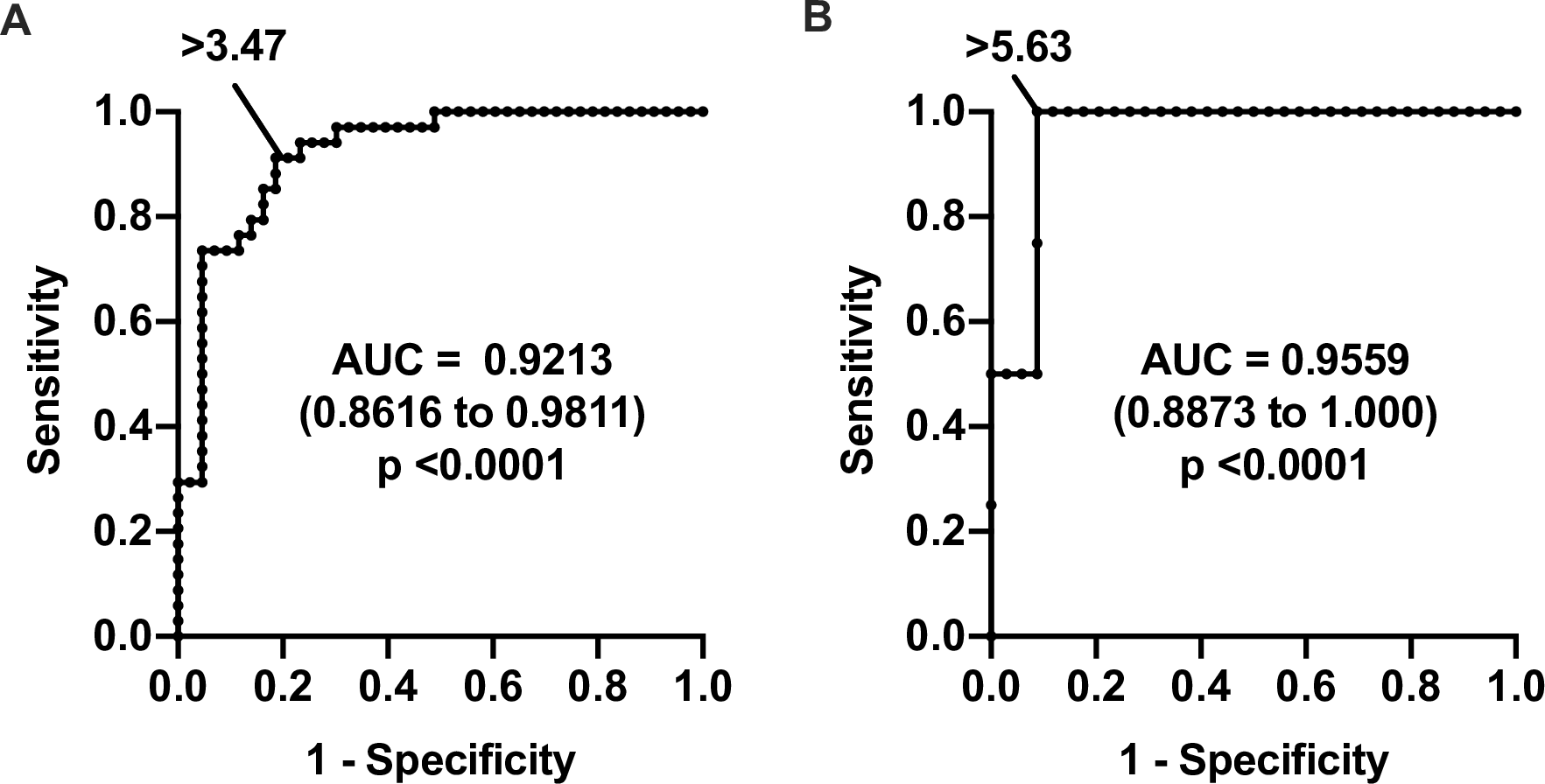

Supplement: S3 Fig — (A) The receiver operating characteristic (ROC) analysis of Accuraseed® immunoanalyzer-based aldosterone-to-renin ratio (CLEIA-ARR) to detect patients with primary aldosteronism (PA) diagnosed only by the captopril challenge test (CCT). Numbers of non-PA and PA samples are 43 and 34, respectively. (B) The ROC analysis of CLEIA-ARR to distinguish unilateral PA patients from non-PA patients. In the analysis shown in this panel, PA was diagnosed by not only CCT. Numbers of non-PA and unilateral PA samples are 34 and 4, respectively. Areas under the curves (AUCs) are shown with 95% confidence intervals in parentheses and p values. Optimal cutoffs are also shown in ng/dL over pg/mL. The data of Basal group, which consists of basal, ambulatory, and before loading samples, and inferior vena cava samples before the adrenocorticotropic hormone loading in the adrenal venous sampling, are used. (TIF) [file pone.0253807.s003.tif]

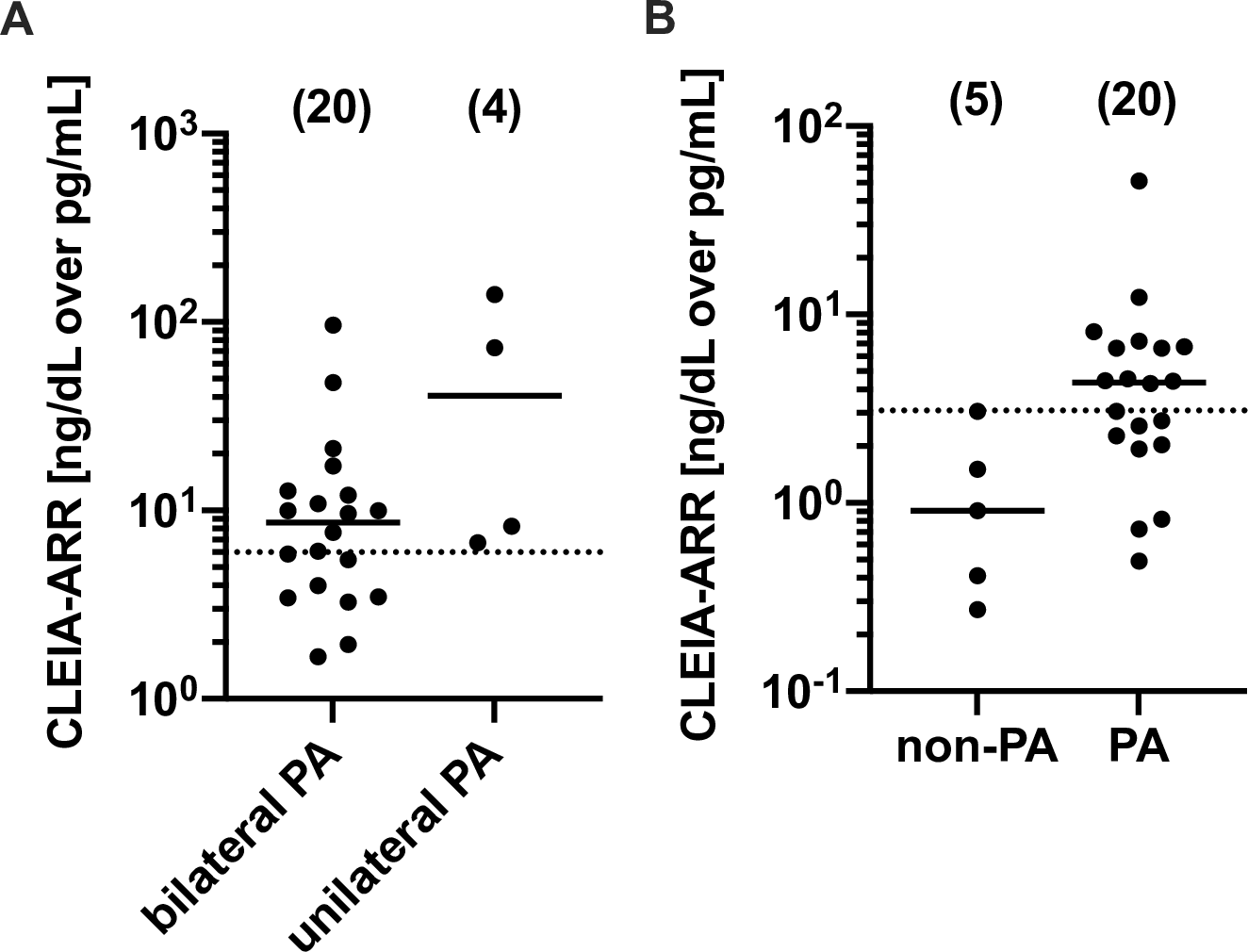

Supplement: S4 Fig — (A) The distributions of Accuraseed® immunoanalyzer-based aldosterone-to-renin ratio (CLEIA-ARR) values in the samples of Basal group, for which AVS was successfully performed, divided by the laterality of PA. (B) The distributions of CLEIA-ARR values in the samples of non-PA and PA patients at 60 min after the loading of captopril challenge test (CCT60). In panels A and B, dotted lines indicate 6 and 3.09 ng/dL over pg/mL of CLEIA-ARR, respectively. Medians are indicated by horizontal bars. Sample numbers are shown in parentheses. (TIF) [file pone.0253807.s004.tif]
