# Supplementary material for: Comparisons of plasma aldosterone and renin data between an automated chemiluminescent immunoanalyzer and conventional radioimmunoassays in the screening and diagnosis of primary aldosteronism
Source: PLoS One. 2021 Jul 9;16(7):e0253807. doi: 10.1371/journal.pone.0253807 (PMC8270132; doi:10.1371/journal.pone.0253807)
Supplement: S1 Table — (DOCX) [file pone.0253807.s005.docx]

**S1 Table. Cross-reactivities of assay kits used in the present study.**

(A) SPAC^®^-S Aldosterone RIA kit

| substances | cross-reactivities | substances | cross-reactivities |
| --- | --- | --- | --- |
| corticosterone | 0.03% | prednisolone | <0.00003% |
| 18-hydroxycorticosterone | 0.01% | spironolactone | 0.0004% |
| cortisol | 0.0002% | 18-hydroxydeoxycorticosterone | 0.04% |
| cortisone | <0.0003% | progesterone | 0.008% |
| deoxycorticosterone | 0.05% | 18-hydroxyprogesterone | 0.005% |
| dexamethasone | <0.00003% | tetrahydrocorticosterone | <0.0003% |

The data claimed by the manufacturer in the product insert are shown.

(B) PRA-FR^®^ RIA kit

| substances | cross-reactivities |
| --- | --- |
| angiotensin II | <0.001% |
| Angiotensin III | <0.001% |

This kit measures the concentrations of angiotensin I generated in samples during incubation. The data claimed by the manufacturer in the product insert are shown.

The cross-reactivities of Accuraseed^®^ Aldosterone kit is available in Table S11. Cross-reactivity in CLEIA of PAC of the following reference: Morimoto R, Ono Y, Tezuka Y, Kudo M, Yamamoto S, Arai T, et al. Rapid Screening of Primary Aldosteronism by a Novel Chemiluminescent Immunoassay. Hypertension. 2017;70: 334-341. doi: 10.1161/HYPERTENSIONAHA.117.09078.
