# Supplementary material for: Comparisons of plasma aldosterone and renin data between an automated chemiluminescent immunoanalyzer and conventional radioimmunoassays in the screening and diagnosis of primary aldosteronism
Source: PLoS One. 2021 Jul 9;16(7):e0253807. doi: 10.1371/journal.pone.0253807 (PMC8270132; doi:10.1371/journal.pone.0253807)
Supplement: S2 Table — (DOCX) [file pone.0253807.s006.docx]

**S2 Table. Numbers and classifications of samples for aldosterone-to-renin ratio analyses.**

| sampling conditions | non-PA | PA | subtotal | group of sampling conditions |
| --- | --- | --- | --- | --- |
| ambulatory | 3 (3) | 0 (0) | 3 (3) | Basal |
| basal | 22 (22) | 9 (9) | 31 (31) |  |
| before loading | 8 (8) | 22 (21) | 30 (29) |  |
| IVC samples before  the ACTH loading in AVS | 0 (0) | 13 (13) | 13 (13) |  |
| Sum of the Basal group | 33 (31) | 44 (30) | 77 (61) |  |
| CCT60 | 5 (5) | 20 (20) | 25 (25) |  |
| Total | 38 (31) | 64 (30) | 102 (61) |  |

The numbers of samples, which were used in statistical analyses on radioimmunoassay-based and Accuraseed^®^ immunoanalyzer-based aldosterone-to-renin ratio (RIA-ARR and CLEIA-ARR, respectively) values, are shown divided by sampling conditions and diagnoses. The numbers of patients, from whom samples used in the above analyses were obtained, are shown in parentheses. Samples obtained from peripheral veins in ambulatory and basal conditions, and before loading in confirmatory tests, and those obtained from the inferior vena cava (IVC) before the adrenocorticotropic hormone (ACTH) loading in the adrenal venous sampling (AVS) were analyzed as the samples of Basal group. Samples obtained from peripheral veins 60 min after the per os administration of 50-mg captopril (CCT60) were also used in the analyses. Patient numbers in sum and total rows are smaller than sums of the rows looked up, where samples of different sampling conditions were obtained from some patients.
