# Supplementary material for: Comparisons of plasma aldosterone and renin data between an automated chemiluminescent immunoanalyzer and conventional radioimmunoassays in the screening and diagnosis of primary aldosteronism
Source: PLoS One. 2021 Jul 9;16(7):e0253807. doi: 10.1371/journal.pone.0253807 (PMC8270132; doi:10.1371/journal.pone.0253807)
Supplement: S3 Table — (DOCX) [file pone.0253807.s007.docx]

**S3 Table. Distributions of PRA and CLEIA-ARC values and relations between them.**

(A) D’Agostino & Pearson tests for normal and log-normal distributions of radioimmunoassay-based plasma renin activity (PRA) and Accuraseed^®^ Renin kit-based plasma active renin concentration (CLEIA-ARC) values

| groups | variables | *n* | normal or log-normal | *K2* | *p* values | probabilities |
| --- | --- | --- | --- | --- | --- | --- |
| All samples | PRA | 140 | normal | 137.3 | <0.0001 | 0% |
|  |  | 140 | log-normal | 17.07 | 0.0002 | 100% |
|  | CLEIA-ARC | 140 | normal | 147.8 | <0.0001 | 0% |
|  |  | 140 | log-normal | 12.65 | 0.0018 | 100% |
| Basal-non-PA | PRA | 41 | normal | 34.26 | <0.0001 | <0.001% |
|  |  | 41 | log-normal | 2.528 | 0.2825* | 100% |
|  | CLEIA-ARC | 41 | normal | 38.59 | <0.0001 | 0% |
|  |  | 41 | log-normal | 1.357 | 0.5073* | 100% |
| Basal-PA | PRA | 44 | normal | 29.10 | <0.0001 | 0.0002% |
|  |  | 44 | log-normal | 2.943 | 0.2296* | 100% |
|  | CLEIA-ARC | 44 | normal | 25.85 | <0.0001 | 0.0005% |
|  |  | 44 | log-normal | 0.2209 | 0.8954* | 100% |
| RStim | PRA | 46 | normal | 65.77 | <0.0001 | 0% |
|  |  | 46 | log-normal | 3.315 | 0.1906* | 100% |
|  | CLEIA-ARC | 46 | normal | 70.31 | <0.0001 | 0% |
|  |  | 46 | log-normal | 1.294 | 0.5237* | 100% |
| Post-SIT | PRA | 9 | normal | 11.93 | 0.0026 | 3.809% |
|  |  | 9 | log-normal | 2.644 | 0.2666* | 96.19% |
|  | CLEIA-ARC | 9 | normal | 21.05 | <0.0001 | 0.9934% |
|  |  | 9 | log-normal | 10.90 | 0.0043 | 99.01% |

Basal-non-PA: Basal group of non-PA samples. Basal-PA: Basal group of PA samples. RStim: Renin stimulation test group. Post-SIT: Post-saline infusion test group.

*Passed normality test.

(B) Linear regression analyses between log-transformed values of CLEIA-ARC and PRA: *x* = log_10_(PRA [ng/mL/h]), *y* = log_10_(CLEIA-ARC [pg/mL])

| groups | regression coefficients | | *SE* | 95% CIs | *p* values | *R^2^* |
| --- | --- | --- | --- | --- | --- | --- |
| Basal-non-PA | slope | 1.199 | 0.03755 | 1.123 to 1.275 | <0.0001 | 0.9632 |
|  | *y*-intercept | 0.8297 | 0.01567 | 0.7980 to 0.8613 |  |  |
| Basal-PA | slope | 1.191 | 0.09344 | 1.003 to 1.380 | <0.0001 | 0.7947 |
|  | *y*-intercept | 0.8593 | 0.04098 | 0.7766 to 0.9420 |  |  |
| RStim | slope | 1.120 | 0.03575 | 1.048 to 1.192 | <0.0001 | 0.9571 |
|  | *y*-intercept | 0.8339 | 0.01497 | 0.8037 to 0.8640 |  |  |
| Post-SIT | slope | 0.7654 | 0.2089 | 0.2715 to 1.259 | 0.0080 | 0.6574 |
|  | *y*-intercept | 0.7276 | 0.1036 | 0.4828 to 0.9725 |  |  |

*SE*: standard error. CI: confidence interval.

Are the slopes equal? *F* = 2.504, the degree of freedom for the numerator (*DFn*) = 3, the degree of freedom for the denominator (*DFd*) = 132, *p* = 0.0620, the pooled slope 1.150.

Are the *y*-intercepts equal? *F* = 0.7383, *DFn* = 3, *DFd* = 135, *p* = 0.5310, the pooled *y*-intercept 0.8417.
