# Supplementary material for: Comparisons of plasma aldosterone and renin data between an automated chemiluminescent immunoanalyzer and conventional radioimmunoassays in the screening and diagnosis of primary aldosteronism
Source: PLoS One. 2021 Jul 9;16(7):e0253807. doi: 10.1371/journal.pone.0253807 (PMC8270132; doi:10.1371/journal.pone.0253807)
Supplement: S4 Table — (DOCX) [file pone.0253807.s008.docx]

**S4 Table. Distributions of RIA-PAC and CLEIA-PAC-1st values and relations between them.**

(A) D’Agostino & Pearson tests for normal and log-normal distributions of radioimmunoassay-based plasma aldosterone concentration (RIA-PAC) values and first values of Accuraseed^®^ Aldosterone kit-based plasma aldosterone concentration (CLEIA-PAC-1st)

| variables | *n* | normal or log-normal | *K2* | *p* values | probabilities |
| --- | --- | --- | --- | --- | --- |
| RIA-PAC | 235 | normal | 224.8 | <0.0001 | 0% |
|  | 235 | log-normal | 43.30 | <0.0001 | 100% |
| CLEIA-PAC-1st | 235 | normal | 222.8 | <0.0001 | 0% |
|  | 235 | log-normal | 46.12 | <0.0001 | 100% |

(B) The linear regression analysis between untransformed values of CLEIA-PAC-1st (*y* [ng/dL]) and RIA-PAC (*x* [ng/dL])

| regression coefficients | | *SE* | 95% CIs | *p* value | *R^2^* |
| --- | --- | --- | --- | --- | --- |
| slope | 0.9931 | 0.008414 | 0.9765 to 1.010 | <0.0001 | 0.9835 |
| *y*-intercept | -4.20 | 12.61 | -29.04 to 20.64 |  |  |

*SE*: standard error. CI: confidence interval.

(C) The Bland-Altman plot between untransformed values of CLEIA-PAC-1st and RIA-PAC

| bias | *SD* | 95% limit of agreement |
| --- | --- | --- |
| -4.716% | 29.87% | -63.27% to 53.83% |

%Difference values are plotted against averages. *SD*: standard deviation.

(D) The non-linear regression analysis between the coefficient of variations of CLEIA-PAC (*y* [%]) and CLEIA-PAC-1st values (*x* [ng/dL]) in samples with CLEIA-PAC-1st values being <20 ng/dL, with a one-phase exponential decay equation: *y* = (Y_0_ - Plateau)^-K^*^x^* + Plateau

| regression coefficients | | 95% CIs | degree of freedom | *R^2^* |
| --- | --- | --- | --- | --- |
| Y_0_ | 133.6 | 76.48 to 236.1 | 134 | 0.3798 |
| Plateau | 7.667 | 4.266 to 10.36 |  |  |
| K | 0.3856 | 0.2506 to 0.5444 |  |  |
| Y_0_ - Plateau | 125.9 | N.A. |  |  |

N.A.: not applicable.
