# Supplementary material for: Comparisons of plasma aldosterone and renin data between an automated chemiluminescent immunoanalyzer and conventional radioimmunoassays in the screening and diagnosis of primary aldosteronism
Source: PLoS One. 2021 Jul 9;16(7):e0253807. doi: 10.1371/journal.pone.0253807 (PMC8270132; doi:10.1371/journal.pone.0253807)
Supplement: S5 Table — (DOCX) [file pone.0253807.s009.docx]

**S5 Table. Intra-assay precision of Accuraseed^®^ Aldosterone kit.**

(A) Aldosterone solutions prepared in the charcoal stripped human serum

| aldosterone concentrations | 6 ng/dL | 9 ng/dL | 12 ng/dL | 15 ng/dL | 40 ng/dL | 80 ng/dL |
| --- | --- | --- | --- | --- | --- | --- |
| 1 | 6.25 | 8.46 | 11.59 | 15.25 | 39.01 | 81.05 |
| 2 | 6.47 | 9.36 | 11.49 | 15.57 | 40.78 | 79.98 |
| 3 | 7.12 | 9.35 | 12.40 | 16.04 | 40.05 | 78.39 |
| 4 | 5.78 | 8.57 | 11.28 | 15.00 | 39.56 | 80.61 |
| 5 | 5.14 | 10.27 | 12.71 | 14.44 | 40.93 | 79.29 |
| mean | 6.15 | 9.20 | 11.89 | 15.26 | 40.07 | 79.86 |
| range | 1.98 | 1.81 | 1.43 | 1.60 | 1.92 | 2.66 |
| *SD* | 0.74 | 0.73 | 0.62 | 0.60 | 0.81 | 1.06 |
| *CV* | 12.1% | 7.9% | 5.2% | 3.9% | 2.0% | 1.3% |

(B) Patient samples with CLEIA-PAC-1st being ≥20 ng/dL

| patients | A | B | C | D | E | F | G | H | I | J |
| --- | --- | --- | --- | --- | --- | --- | --- | --- | --- | --- |
| 1 | 19.63 | 21.94 | 27.02 | 34.73 | 41.66 | 48.16 | 62.12 | 77.54 | 93.54 | 129.3 |
| 2 | 20.63 | 23.21 | 28.36 | 35.61 | 43.16 | 47.29 | 61.22 | 75.43 | 90.42 | 129.4 |
| 3 | 19.46 | 23.60 | 27.21 | 35.58 | 41.88 | 47.65 | 61.55 | 76.46 | 92.63 | 129.0 |
| mean | 19.91 | 22.92 | 27.53 | 35.31 | 42.23 | 47.70 | 61.63 | 76.48 | 92.20 | 129.2 |
| range | 1.17 | 1.66 | 1.34 | 0.88 | 1.50 | 0.87 | 0.90 | 2.11 | 3.12 | 0.46 |
| *SD* | 0.63 | 0.87 | 0.73 | 0.50 | 0.81 | 0.44 | 0.46 | 1.06 | 1.61 | 0.24 |
| *CV* | 3.2% | 3.8% | 2.6% | 1.4% | 1.9% | 0.92% | 0.74% | 1.4% | 1.7% | 0.18% |

Aldosterone concentrations are shown in ng/dL. *SD*: standard deviation. *CV*: coefficient of variation. CLEIA-PAC-1st: the first value of Accuraseed^®^ Aldosterone kit-based plasma aldosterone concentration.
