# Supplementary material for: Comparisons of plasma aldosterone and renin data between an automated chemiluminescent immunoanalyzer and conventional radioimmunoassays in the screening and diagnosis of primary aldosteronism
Source: PLoS One. 2021 Jul 9;16(7):e0253807. doi: 10.1371/journal.pone.0253807 (PMC8270132; doi:10.1371/journal.pone.0253807)
Supplement: S6 Table — (DOCX) [file pone.0253807.s010.docx]

**S6 Table. Relations between CLEIA-PAC-final and RIA-PAC values.**

(A) D’Agostino & Pearson tests for normal and log-normal distributions of the final values of Accuraseed^®^ Aldosterone kit-based plasma aldosterone concentration (CLEIA-PAC-final)

| variable | *n* | normal or log-normal | *K2* | *p* values | probabilities |
| --- | --- | --- | --- | --- | --- |
| CLEIA-PAC-final | 221 | normal | 204.7 | <0.0001 | 0% |
|  | 221 | log-normal | 43.07 | <0.0001 | 100% |

(B) The linear regression analysis between untransformed values of CLEIA-PAC-final (*y* [ng/dL]) and RIA-PAC (*x* [ng/dL])

| regression coefficients | | *SE* | 95% CIs | *p* value | *R^2^* |
| --- | --- | --- | --- | --- | --- |
| slope | 0.9931 | 0.008703 | 0.9759 to 1.010 | <0.0001 | 0.9835 |
| *y*-intercept | -3.980 | 13.45 | -30.48 to 22.52 |  |  |

*SE*: standard error. CI: confidence interval.

(C) The Bland-Altman plot between untransformed values of CLEIA-PAC-final and RIA-PAC

| bias | *SD* | 95% limit of agreement |
| --- | --- | --- |
| -0.07383% | 27.13% | -53.26% to 53.11% |

%Difference is plotted against the average. *SD*: standard deviation.
