# Supplementary material for: Comparisons of plasma aldosterone and renin data between an automated chemiluminescent immunoanalyzer and conventional radioimmunoassays in the screening and diagnosis of primary aldosteronism
Source: PLoS One. 2021 Jul 9;16(7):e0253807. doi: 10.1371/journal.pone.0253807 (PMC8270132; doi:10.1371/journal.pone.0253807)
Supplement: S7 Table — (DOCX) [file pone.0253807.s011.docx]

**S7 Table. Distributions of RIA-PAC and CLEIA-PAC values and relations between them.**

(A) D’Agostino & Pearson tests for normal and log-normal distributions of radioimmunoassay-based plasma aldosterone concentration (RIA-PAC) and Accuraseed^®^ Aldosterone kit-based plasma aldosterone concentration (CLEIA-PAC) values in five groups

| groups | variables | *n* | normal or log-normal | *K2* | *p* values | probabilities |
| --- | --- | --- | --- | --- | --- | --- |
| Basal-non-PA | RIA-PAC | 34 | normal | 6.028 | 0.0491 | 1.908% |
|  |  | 34 | log-normal | 0.2268 | 0.8928* | 98.09% |
|  | CLEIA-PAC | 34 | normal | 15.11 | 0.0005 | 0.05091% |
|  |  | 34 | log-normal | 2.433 | 0.2962* | 99.95% |
| Basal-PA | RIA-PAC | 50 | normal | 61.01 | <0.0001 | 0% |
|  |  | 50 | log-normal | 16.01 | 0.0003 | 100% |
|  | CLEIA-PAC | 50 | normal | 52.07 | <0.0001 | <0.0001% |
|  |  | 50 | log-normal | 12.35 | 0.0021 | 100% |
| ASup | RIA-PAC | 39 | normal | 33.86 | <0.0001 | 0.002877% |
|  |  | 39 | log-normal | 2.749 | 0.2530* | 100% |
|  | CLEIA-PAC | 39 | normal | 63.43 | <0.0001 | <0.0001% |
|  |  | 39 | log-normal | 20.34 | <0.0001 | 100% |
| AStim | RIA-PAC | 40 | normal | 48.62 | <0.0001 | <0.0001% |
|  |  | 40 | log-normal | 4.825 | 0.0896* | 100% |
|  | CLEIA-PAC | 40 | normal | 42.04 | <0.0001 | <0.0001% |
|  |  | 40 | log-normal | 6.692 | 0.0352 | 100% |
| AdV | RIA-PAC | 58 | normal | 23.83 | <0.0001 | 0% |
|  |  | 58 | log-normal | 5.786 | 0.0554* | 100% |
|  | CLEIA-PAC | 58 | normal | 22.18 | <0.0001 | 0% |
|  |  | 58 | log-normal | 2.240 | 0.3263* | 100% |

CLEIA-PAC values mean CLEIA-PAC-final values in this table. Basal-non-PA: Basal group of non-PA samples. Basal-PA: Basal group of PA samples. ASup: Aldosterone suppression test group. AStim: Aldosterone stimulation test group. AdV: Adrenal vein sample group.

*Passed normality test.

(B) Linear regression analyses between log-transformed values of CLEIA-PAC and RIA-PAC in five groups: *x* = log_10_(RIA-PAC [ng/dL]), *y* = log_10_(CLEIA-PAC [ng/dL])

| groups (complexes) | regression coefficients | | *SE* | 95% CIs | *p* values | *R^2^* |
| --- | --- | --- | --- | --- | --- | --- |
| Basal-non-PA  (A) | slope | 0.6137 | 0.08891 | 0.4326 to 0.7948 | <0.0001 | 0.5982 |
|  | *y*-intercept | 0.4498 | 0.09082 | 0.2648 to 0.6348 |  |  |
| Basal-PA  (B) | slope | 0.9619 | 0.04939 | 0.8626 to 1.061 | <0.0001 | 0.8877 |
|  | *y*-intercept | 0.02614 | 0.06134 | -0.09720 to 0.1495 |  |  |
| ASup  (A) | slope | 0.6174 | 0.08765 | 0.4398 to 0.7950 | <0.0001 | 0.5729 |
|  | *y*-intercept | 0.4164 | 0.08734 | 0.2394 to 0.5934 |  |  |
| AStim  (B) | slope | 0.8876 | 0.04435 | 0.7978 to 0.9774 | <0.0001 | 0.9133 |
|  | *y*-intercept | 0.1322 | 0.06786 | -0.005158 to 0.2696 |  |  |
| AdV  (B) | slope | 0.9839 | 0.02099 | 0.9419 to 1.026 | <0.0001 | 0.9751 |
|  | *y*-intercept | 0.02059 | 0.06210 | -0.1038 to 0.1450 |  |  |

*SE*: standard error. CI: confidence interval.

Among all groups:

Are the slopes equal? *F* = 9.046, the degree of freedom for the numerator (*DFn*) = 4, the degree of freedom for the denominator (*DFd*) = 211, *p* <0.001.

In the complex A (the Basal-non-PA and ASup groups):

Are the slopes equal? *F* = 0.0009031, *DFn* = 1, *DFd* = 69, *p* = 0.9761, the pooled slope 0.6154.

Are the *y*-intercepts equal? *F* = 1.326, *DFn* = 1, *DFd* = 70, *p* = 0.2534, the pooled *y*-intercept 0.4323.

In the complex B (the Basal-PA, AStim, and AdV groups):

Are the slopes equal? *F* = 1.692, *DFn* = 2, *DFd* = 142, *p* = 0.1878, the pooled slope 0.9704.

Are the *y*-intercepts equal? *F* = 1.305, *DFn* = 2, *DFd* = 144, *p* = 0.2743, the pooled *y*-intercept 0.03103.
