# Supplementary material for: Comparisons of plasma aldosterone and renin data between an automated chemiluminescent immunoanalyzer and conventional radioimmunoassays in the screening and diagnosis of primary aldosteronism
Source: PLoS One. 2021 Jul 9;16(7):e0253807. doi: 10.1371/journal.pone.0253807 (PMC8270132; doi:10.1371/journal.pone.0253807)
Supplement: S8 Table — (DOCX) [file pone.0253807.s012.docx]

**S8 Table. Distributions of RIA-ARR and CLEIA-ARR values and the relation between them.**

(A) D’Agostino & Pearson tests for normal and log-normal distributions of radioimmunoassay-based aldosterone-to-renin ratio (RIA-ARR) and Accuraseed^®^ immunoanalyzer-based aldosterone-to-renin ratio (CLEIA-ARR) values

| variables | *n* | normal or log-normal | *K2* | *p* values | probabilities |
| --- | --- | --- | --- | --- | --- |
| RIA-ARR | 102 | normal | 168.4 | <0.0001 | 0% |
|  | 102 | log-normal | 3.731 | 0.1548* | 100% |
| CLEIA-ARR | 102 | normal | 163.5 | <0.0001 | 0% |
|  | 102 | log-normal | 3.818 | 0.1482* | 100% |

The data of the samples of Basal group and CCT60 samples were analyzed together.

*Passed normality test.

(B) The linear regression analysis between log-transformed values of CLEIA-ARR and RIA-ARR: *x* = log_10_(RIA-ARR [ng/dL over ng/mL/h]), *y* = log_10_(CLEIA-ARR [ng/dL over pg/mL])

| regression coefficients | | *SE* | 95% CIs | *p* value | *R^2^* |
| --- | --- | --- | --- | --- | --- |
| slope | 1.050 | 0.03720 | \| 0.9760 to 1.124 \| \| --- \| | <0.0001 | 0.8884 |
| *y*-intercept | -0.8657 | 0.05028 | -0.9655 to -0.7660 |  |  |

*SE*: standard error. CI: confidence interval.
