# Supplementary material for: Comparisons of plasma aldosterone and renin data between an automated chemiluminescent immunoanalyzer and conventional radioimmunoassays in the screening and diagnosis of primary aldosteronism
Source: PLoS One. 2021 Jul 9;16(7):e0253807. doi: 10.1371/journal.pone.0253807 (PMC8270132; doi:10.1371/journal.pone.0253807)
Supplement: S9 Table — (DOCX) [file pone.0253807.s013.docx]

**S9 Table. Distributions of RIA-ARR and CLEIA-ARR values and their relations in two sample groups.**

(A) D’Agostino & Pearson tests for normal distribution of log-transformed values of radioimmunoassay-based aldosterone-to-renin ratio (RIA-ARR) and Accuraseed^®^ immunoanalyzer-based aldosterone-to-renin ratio (CLEIA-ARR) in the samples of Basal group and CCT60 samples.

| groups | variables | *n* | *K2* | *p* values |
| --- | --- | --- | --- | --- |
| Basal | RIA-ARR | 77 | 3.622 | 0.1635* |
|  | CLEIA-ARR | 77 | 3.963 | 0.1378* |
| CCT60 | RIA-ARR | 25 | 0.2585 | 0.8787* |
|  | CLEIA-ARR | 25 | 0.5963 | 0.7422* |

The data of the samples of Basal group and CCT60 samples were analyzed separately.

*Passed normality test. CCT60: 60 min after the loading of captopril challenge test.

(B) Linear regression analyses between log-transformed values of CLEIA-ARR and RIA-ARR in the samples of Basal group and CCT60 samples: *x* = log_10_(RIA-ARR [ng/dL over ng/mL/h]), *y* = log_10_(CLEIA-ARR [ng/dL over pg/mL]).

| groups | regression coefficients | | *SE* | 95% CIs | *p* values | *R^2^* |
| --- | --- | --- | --- | --- | --- | --- |
| Basal | slope | 1.039 | 0.04060 | 0.9576 to 1.119 | <0.0001 | 0.8972 |
|  | *y*-intercept | -0.8546 | 0.05545 | -0.9650 to -0.7441 |  |  |
| CCT60 | slope | 1.101 | 0.09322 | 0.9083 to 1.294 | <0.0001 | 0.8585 |
|  | *y*-intercept | -0.9141 | 0.1217 | -1.171 to -0.6676 |  |  |

*SE*: standard error. CI, confidence interval.

Are the slopes equal? *F* = 0.4234, the degree of freedom for the numerator (*DFn*) = 1, the degree of freedom for the denominator (*DFd*) = 98, *p* = 0.5168, the pooled slope 1.050.

Are the *y*-intercepts equal? *F* = 0.09662, *DFn* = 1, *DFd* = 99, *p* = 0.7566,

the pooled *y*-intercept -0.8662.
