# Supplementary material for: Comparisons of plasma aldosterone and renin data between an automated chemiluminescent immunoanalyzer and conventional radioimmunoassays in the screening and diagnosis of primary aldosteronism
Source: PLoS One. 2021 Jul 9;16(7):e0253807. doi: 10.1371/journal.pone.0253807 (PMC8270132; doi:10.1371/journal.pone.0253807)
Supplement: S11 Table — (DOCX) [file pone.0253807.s015.docx]

**S11 Table. The relation between CLEIA-ARC and PRA values.**

| regression coefficients | | *SE* | 95% CIs | *p* value | *R^2^* |
| --- | --- | --- | --- | --- | --- |
| slope | 9.626 | 0.1698 | 9.291 to 9.962 | <0.0001 | 0.9588 |
| *y*-intercept | -1.956 | 0.3743 | -2.696 to -1.216 |  |  |

The result of linear regression analysis between untransformed values of Accuraseed^®^ Renin kit-based plasma active renin concentration (CLEIA-ARC) and radioimmunoassay-based plasma renin activity (PRA) is shown. *x* = PRA [ng/mL/h], *y* = CLEIA-ARC [pg/mL], *n* = 140. *SE*: standard error. CI: confidence interval.
