# Supplementary material for: Comparisons of plasma aldosterone and renin data between an automated chemiluminescent immunoanalyzer and conventional radioimmunoassays in the screening and diagnosis of primary aldosteronism
Source: PLoS One. 2021 Jul 9;16(7):e0253807. doi: 10.1371/journal.pone.0253807 (PMC8270132; doi:10.1371/journal.pone.0253807)
Supplement: S12 Table — (DOCX) [file pone.0253807.s016.docx]

**S12 Table. Descriptive statistics of CLEIA-PAC values.**

| complexes | *n* | minimum | 25th percentile | median | 75th percentile | maximum | range |
| --- | --- | --- | --- | --- | --- | --- | --- |
| A | 73 | 6.04 | 8.21 | 9.89 | 14.21 | 42.33 | 36.29 |
| B | 148 | 6.02 | 15.40 | 32.93 | 333.5 | 9410 | 9404 |

Accuraseed^®^ Aldosterone kit-based plasma aldosterone concentration (CLEIA-PAC) values are shown in two complexes: the complex A consisting of the Basal-non-PA and Aldosterone suppression test groups, and the complex B consisting of the Basal-PA, Aldosterone stimulation test, and Adrenal vein sample groups. The unit of CLEIA-PAC: ng/dL.
