# Supplementary material for: Comparisons of plasma aldosterone and renin data between an automated chemiluminescent immunoanalyzer and conventional radioimmunoassays in the screening and diagnosis of primary aldosteronism
Source: PLoS One. 2021 Jul 9;16(7):e0253807. doi: 10.1371/journal.pone.0253807 (PMC8270132; doi:10.1371/journal.pone.0253807)
Supplement: S14 Table — (DOCX) [file pone.0253807.s018.docx]

**S14 Table. Difference of CLEIA-ARR values between lateralities of primary aldosteronism.**

| laterality | *n* | median [ng/dL over pg/mL] | Mann-Whitney *U* | *p* value |
| --- | --- | --- | --- | --- |
| bilateral | 20 | 8.65 | 22 | 0.1826 |
| unilateral | 4 | 40.66 |  |  |

The result of Mann-Whitney U test of Accuraseed^®^ immunoanalyzer-based aldosterone-to-renin ratio (CLEIA-ARR) values of samples in the Basal group between bilateral and unilateral primary aldosteronism is shown. Samples from patients with primary aldosteronism, for whom the laterality of disease was determined by the adrenal venous sampling, were analyzed.
